# Non-Causal Effects of Asthma on COVID-19 Susceptibility and Severity

**DOI:** 10.3389/fgene.2021.762697

**Published:** 2022-01-10

**Authors:** Li-Juan Qiu, Kang-Jia Yin, Gui-Xia Pan, Jing Ni, Bin Wang

**Affiliations:** ^1^ Department of Epidemiology and Biostatistics, School of Public Health, Anhui Medical University, Hefei, China; ^2^ Inflammation and Immune Mediated Diseases Laboratory of Anhui Province, Hefei, China; ^3^ Medical Insurance Office, The Fourth Affiliated Hospital of Anhui Medical University, Hefei, China

**Keywords:** asthma, moderate-to-severe asthma, COVID-19, susceptibility, severity, Mendelian randomization

## Abstract

**Background:** Asthma is observationally associated with an increased risk of COVID-19, but the causality remains unclear. We aim to determine whether there is a casual role of asthma in susceptibility to SARS-CoV-2 infection or COVID-19 severity.

**Methods:** Instrumental variables (IVs) for asthma and moderate-to-severe asthma were obtained from publicly available summary statistics from the most recent and largest genome-wide association study (GWAS), including 394 283 and 57 695 participants of European ancestry, respectively. The corresponding data for COVID-19 susceptibility, hospitalization and severe-disease were derived from the COVID-19 Host Genetics Initiative GWAS meta-analysis of up to 1 683 768 individuals of European descent. Causality was inferred between correlated traits by Mendelian Randomization analyses. Inverse-variance weighted method was used as the primary MR estimates and multiple alternate approaches and several sensitivity analyses were also conducted.

**Results:** Our MR analysis revealed no causal effects of asthma on COVID-19 susceptibility, hospitalization or severe disease, with odds ratio (OR) of 0.994 (95% CI: 0.962–1.027), 1.020 (95% CI: 0.955–1.089), and 0.929 (95% CI: 0.836–1.032), respectively. Furthermore, using genetic variants for moderate-to-severe asthma, a similar pattern of results was observed for COVID-19 susceptibility (OR: 0.988, 95% CI: 0.946–1.031), hospitalization (OR: 0.967, 95% CI: 0.906–1.031), and severe disease (OR: 0.911, 95% CI: 0.823–1.009). The association of asthma and moderate-to-severe asthma with COVID-19 was overall robust to sensitivity analyses.

**Conclusion:** Genetically predicted asthma was not associated with susceptibility to, or severity of, COVID-19 disease, indicating that asthma is unlikely to be a causal factor in the development of COVID-19.

## Introduction

Coronavirus disease 2019 (COVID-19) is a global pandemic infectious disease caused by the severe acute respiratory syndrome coronavirus 2 (SARS-CoV-2), and has posed a serious threat to public health and socioeconomic stability worldwide since December 2019 (Liu et al., 2020; Wiersinga et al., 2020). As of July 27, 2021, up to 194 million people with confirmed COVID-19, and 2 million deaths have been reported globally according to the World Health Organization. The global clinical presentation of COVID-19 indicates that there was a high level of heterogeneity in the severity of illness, ranging from asymptomatic and mild symptoms to severe illness and death (Chen et al., 2021), resulting from coronavirus-host interactions (de Wilde et al., 2018). Hence, it is extremely important to identifying fragile populations with higher susceptibility and worse prognosis of COVID-19 (Liu et al., 2021). Currently, a series of co-morbidities including hypertension, type 2 diabetes, chronic kidney disease, and cardiovascular diseases exhibited strong and consistent evidence for association with COVID-19 disease severity and progression (Chen et al., 2020; Richardson et al., 2020; Yang et al., 2020; Zheng et al., 2020).

Asthma is the commonest chronic respiratory disease, which characterized by airway inflammation and hyperreactivity (Alwarith et al., 2020). Considering that patients with asthma have impaired antiviral immune responses against virus infection and a tendency of exacerbation elicited by common respiratory viruses, it seems that pre-existing asthma has a potential influence on SARS-CoV-2 susceptibility and pathological process (Liu et al., 2020). Accordingly, the European Academy of Allery and Clinical Immunology (EAACI) Section on Pediatrics (Brough et al., 2020) and US Centers for Disease Control and Prevention (CDC) (Centers for Disease Control and Prevention, 2020) declared that individuals with asthma could be at an increased risk of COVID-19. This was supported by several clinical and epidemiological studies of different sizes from multiple counties (Azar et al., 2020; Butler et al., 2020; Garg et al., 2020; Goyal et al., 2020; Hernández-Galdamez et al., 2020; Jehi et al., 2020; Kalyanaraman Marcello et al., 2020; Richardson et al., 2020; Ricoca Peixoto et al., 2020; Williamson et al., 2020). For instance, a population-based cohort study of adults hospitalized with laboratory-confirmed COVID-19 in 14US states reported that asthma was one of the most common comorbidities (17% prevalence) (Garg et al., 2020). Besides, a cohort (clinical) study using an unprecedented scale of 17 million patient’s detailed primary care records in the United Kingdom demonstrated that individuals with asthma had a higher risk of severe COVID-19 (Williamson et al., 2020). Nonetheless, any causal link between asthma and COVID-19 susceptibility and severity remains unclear as conventional observational studies can always be hampered by residual confounding and/or reverse causation bias (Vaucher et al., 2018).

Mendelian randomization (MR) is a genetic epidemiological approach that utilizing genetic variants as instrumental variables (IVs), to appraise causality between exposure and disease outcomes when this relationship cannot be directly assessed by clinical trials (Burgess et al., 2015a; Dan et al., 2020). Since germline genetic variants are randomly set at conception and established well before onset of disease, MR analysis minimizes issues of confounding by lifestyle and environmental factors and avoids reverse causal bias. In a two-sample MR design, instrument-exposure and instrument-outcome can be extracted from summary statistics of separate nonoverlapping samples, improving effect size estimation and statistical power. In present study, we applied a two-sample MR approach to infer causal effects of asthma phenotypes on COVID-19 susceptibility and severity.

## Materials and Methods

### Study Design and Data Sources

A two sample MR analysis was conducted to determine whether genetic predisposition to asthma and moderate-to-severe asthma is causally linked to COVID-19 susceptibility and severity. The MR approach relies on the following three assumptions (Supplementary Figure S1): first, the genetic variants must be powerfully correlated with the exposure (here, asthma); second, the genetic variants must be unrelated to any confounding factors that are associated with the outcome (here, COVID-19 susceptibility and severity); Lastly, the genetic variants must affect the outcome only through exposure factors rather than via alternative ways (also known as an absence of horizontal pleiotropy) (Pierce and Burgess, 2013). This approach based on the largest genome-wide association study (GWAS) summary statistics from published available data, which were summarized in Supplementary Table S1. All these data herein were publicly available, and thus further ethical approval was not required in this work.

### Asthma

Genetic instruments for asthma derived from Zhu et al. (2019) large-scale genome-wide association study (GWAS) for cross-trait analysis between asthma and mental health disorders, in which the largest GWAS for asthma using phenotype data provided for United Kingdom Biobank participants of European ancestry. Full details of United Kingdom Biobank cohort are available elsewhere (Sudlow et al., 2015) and in Supplementary Table S1. Briefly, a total of 46 802 cases with asthma, and 347 481 controls were involved.

### Moderate-to-Severe Asthma

Genetic instruments for moderate-to-severe asthma were identified using the publicly available GWAS repository on moderate-to-severe asthma (Shrine et al., 2019), which combined summary statistics from two United Kingdom cohorts (the Genetics of Asthma Severity and Phenotypes [GASP] initiative and the Unbiased BIOmarkers in PREDiction of respiratory disease outcomes [U-BIOPRED] project) with GWAS for moderate-to-severe asthma performed in United Kingdom Biobank, with 10 549 cases and 47 146 controls of European ancestry as shown in Supplementary Table S1.

### COVID-19

The instrumental variables for COVID-19 were retrieved from the largest GWAS meta-analysis of COVID-19 (round 5) by the COVID-19 Host Genetics Initiative (The COVID-19 Host Genetics Initiative, 2020) (Supplementary Table S1), which was shared publicly on January 18, 2021. For all outcomes, a confirmed case of COVID-19 was defined as testing positive for SARS-CoV-2 infection by RNA reverse transcription polymerase chain reaction (RT-PCR), serological testing, or clinician diagnosis by chart review or International Classification of Diseases coding or self-reporting. To avoid the ethnic heterogeneity of genetic association, we restricted analysis to participants of European ancestry only. For COVID-19 susceptibility, we used a susceptibility phenotype that compared confirmed COVID-19 cases (N = 38 984) with population controls (N = 1 644 784), referred to as C2 in the COVID-19 HGI documentation. For COVID-19 severity, we used two approaches. The first, we used a hospitalized phenotype in which cases were defined as hospitalized COVID-19 patients (N = 9 986) and population controls (N = 1 877 672), referred to as B2 in the COVID-19 HGI documentation. The other, we used a severe-disease phenotype in which cases were confirmed as “very severe respiratory” COVID-19 (N = 5 101) who required respiratory support (including intubation, continuous positive airway pressure, bilevel positive pressure, continuous external pressure or high-flow nasal cannula), and controls were general population samples (N = 1 383 241), referred to as A2 in the COVID-19 HGI documentation.

### Selection of Instrumental Variables

Independent GWAS-derived exposure-associated genetic instruments, a significance threshold of *p* < 5 × 10^−8^ and not in linkage disequilibrium (*r* ^2^ < 0.001) with each other, were initially selected as the genetic instruments. The summary statistics of these initial genetic instruments were retrieves from the outcome trait GWAS summary. By default, if the specified SNPs were not present in the outcome GWAS summary, then a suitable proxy variant that was in high linkage disequilibrium (*r* ^2^ > 0.6 in European population) and available in both the exposure and outcome GWAS summary were selected as the genetic instrument to instead of the initial one. If no proxies could be identified, the genetic instrument was removed. Then, we harmonized the effect sizes for the SNPs on the exposure (asthma and moderate-to-severe asthma) and the outcome (COVID-19 susceptibility, hospitalization and severe disease) data, and excluded palindromic SNPs with intermediate allele frequency higher than 0.4 from MR analysis. We computed *R* ^2^ to estimate the proportion of variance in the exposure which were explained by the genetic instruments. Meanwhile, in order to quantitatively verify instrument strength, F-statistics for each instrumental variables individually and cumulatively were calculated via the formula F-statistic = *R* ^2^ × (SampleSize-2)/(1-R^2^) (Palmer et al., 2012). If instrumental variables with a F-statistic much than 10, the association was regarded as strong enough to avoid the weak instrument bias (Sanderson et al., 2021).

### Statistical Analysis

We utilized several MR methods to perform MR analyses, including inverse-variance weighted (IVW) (Burgess et al., 2013; Burgess et al., 2015b), weighted-median (Bowden et al., 2016), weighted mode (Hartwig et al., 2017) and MR Egger (Bowden et al., 2015) approaches. IVW method was applied for main MR analysis, which uses a meta-analysis approach to get the overall effect of the exposure on the outcome. Specifically, the weighted-median method, which can provide an unbiased estimate even when up to at 50% of the weight in the analysis arises from invalid IVs (Bowden et al., 2016), and MR-Egger regression, which is robust even if all variants are invalid were tested as complementary methods to evaluate the robustness of the causal estimate.

To detect for presence of unmeasured pleiotropy, we conducted heterogeneity test, MR-Egger intercept test (Bowden et al., 2015), and global test of MR-PRESSO (Verbanck et al., 2018). The heterogeneity of individual genetic instruments was estimated by Cochran Q test. A non-zero intercept (*p* < 0.05) in MR-Egger indicates that directional horizontal pleiotropy is driving the causal estimate (Bowden et al., 2015). Global test of MR-PRESSO was used to identify outlying SNPs that are potentially horizontally pleiotropic (Verbanck et al., 2018). Moreover, leave-one-out permutation analyses were applied in IVW models, where the TSMR is performed again but removing each SNP in turn to identify potentially influential SNPs (Dan et al., 2020). In addition, we calculated the statistical power of our study using the mRnd (http://cnsgenomics.com/shiny/mRnd), proposed by Brion et al. (2013).

All the statistical analyses were carried out using RStudio (version 1.2.5019) with R packages TwoSampleMR, and MR Pleiotropy Residual Sum and Outlier (MR-PRESSO). Results for causal effects were presented as odds ratio (OR) with corresponding 95% CIs. A two-tailed *p* < 0.05 was considered as statistically significant in all estimates.

## Results

### Genetic Instruments

Of the 161 genetic variants from the asthma GWAS dataset, only 149, 144 and 148 SNPs, explaining 2.35, 2.27 and 2.34% of the variance of asthma, were finally included in the GWAS of COVID-19 susceptibility, hospitalization and severe disease, respectively. Characteristics of asthma-associated SNPs and their associated estimates with COVID-19 are presented in Supplementary Table S2. The F-statistic values for individual SNPs ranged from 26 to 294, with means of 62 for asthma. Power calculations for the MR analyses (Supplementary Table S3) indicated greater than 80% statistical power to detect an OR bigger than 1.094, 1.061 and 1.256 for asthma on COVID-19 susceptibility, hospitalization and severe disease, respectively.

As an additional analysis, we obtained 24 independent SNPs as IVs for moderate-to-severe asthma at genome-wide significance. These SNPs were broadly distinct from the SNPs associated with asthma. For one SNP (rs9273410) associated with moderate-to-severe asthma, no proxy was found. Moreover, one palindromic SNPs (rs1131017) with intermediate allele frequencies was removed. Thus, 22 SNPs were finally taken as valid IVs to perform MR analysis (Supplementary Table S2), which explained about 2.45% variance of moderate-to-severe asthma. F-statistic values for moderate-to-severe asthma were more than 10 (ranged from 33 to 135). Power analyses (Supplementary Table S3) revealed that our MR analyses had 80% power to detect an OR of 1.092, 1.059 and 1.250 for moderate-to-severe asthma on COVID-19 susceptibility, hospitalization and severe disease, respectively.

### Two-Sample MR Analysis

**FIGURE 1:**
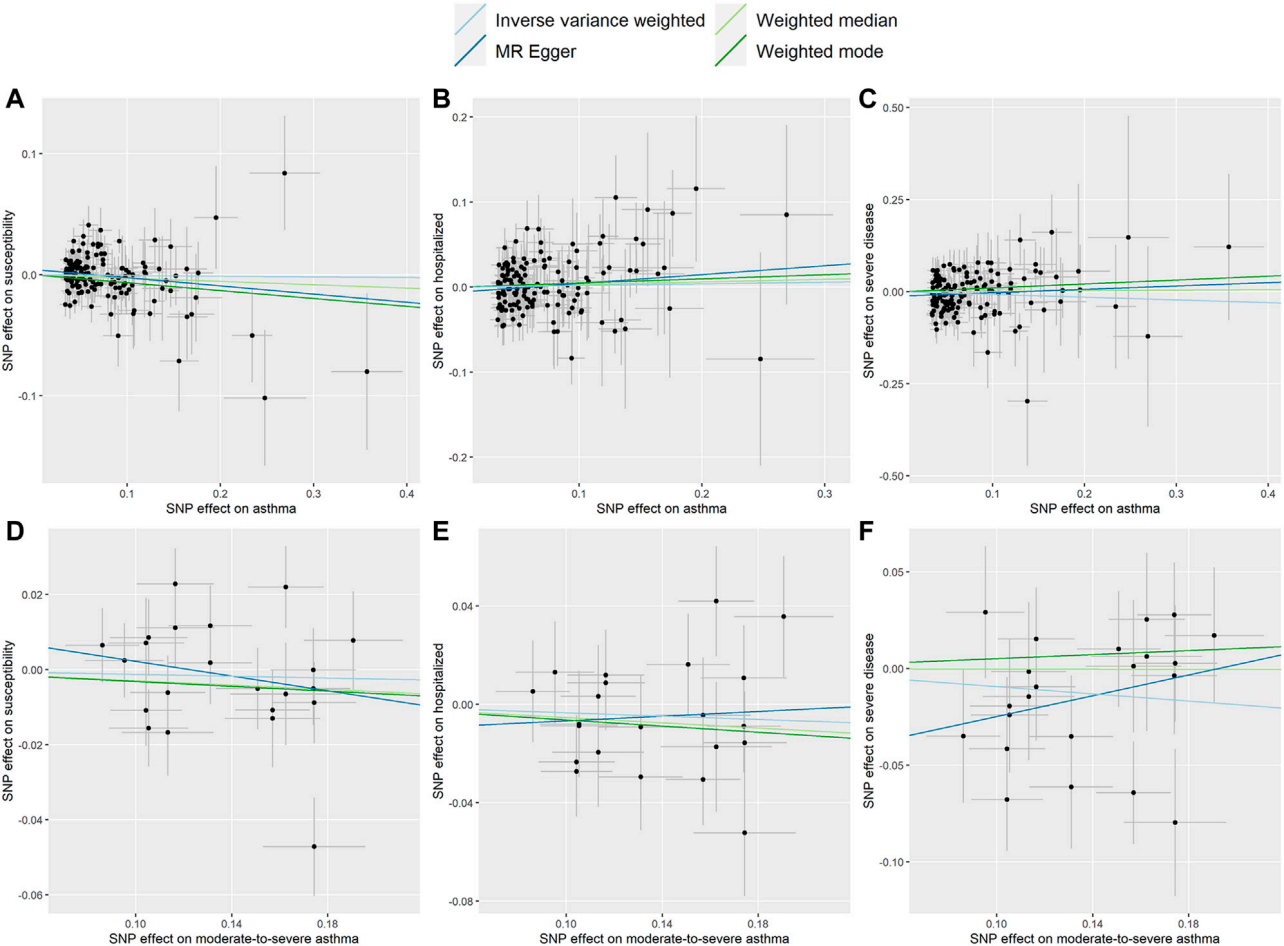
Scatter plots for effect sizes of SNPs for asthma and those for COVID-19 susceptibility, hospitalization and severe disease. **(A–C)** Scatter plots for asthma SNPs; **(D–F)** Scatter plots for moderate-to-severe asthma SNPs. The *x*-axis represents the effect size of SNPs on asthma and moderate-to-severe asthma; the *y*-axis represents the effect size of SNPs on COVID-19 susceptibility, hospitalization and severe disease.

Two-sample MR analysis revealed that asthma was not causally related to COVID-19. The ORs of COVID-19 susceptibility, hospitalization and severe disease per log-odds increment in genetically determined risk of asthma in the primary analyses were 0.994 (95% CI, 0.962–1.027, *p* = 0.727), 1.020(95% CI, 0.955–1.089, *p* = 0.562), and 0.929 (95% CI, 0.836–1.032, *p* = 0.167), respectively. Likewise, the weighted median, weighted mode and MR-Egger methods generated consistent effect estimates compared with IVW (Table 1 and Figure 1). MR-Egger intercept tests, as well as MR-PRESSO global tests, were insignificant (all the *p* value > 0.05, Table 2), implying the absence of horizontal pleiotropy. Similarly, no heterogeneity among individual SNPs was detected from Cochran’s Q statistics (Table 2). Additionally, the forest plot, funnel plot, and leave-one-out plot provided extra support that there was no single outlier influence the estimated causal effect (Supplementary Figures S2–4).

**TABLE 1:** Causal relationships of asthma on COVID-19 estimated by approach of IVW, MR-Weighted median, MR-Weighted mode and MR Egger.

Exposure	Outcome	nSNPs	IVW		MR-weighted median		MR-weighted mode		MR Egger	
			OR (95% CI)	*p*-value	OR (95% CI)	*p*-value	OR (95% CI)	*p*-value	OR (95% CI)	*p*-value
asthma	Susceptibility	149	0.994 (0.962–1.027)	0.727	0.973 (0.926–1.022)	0.278	0.937 (0.858–1.023)	0.149	0.935 (0.866–1.009)	0.085
	Hospitalization	144	1.020 (0.955–1.089)	0.562	1.031 (0.930–1.144)	0.558	1.050 (0.823–1.342)	0.694	1.110 (0.948–1.300)	0.197
	Severe disease	148	0.929 (0.836–1.032)	0.167	1.015 (0.863–1.194)	0.856	1.111 (0.794–1.554)	0.540	1.094 (0.856–1.400)	0.473
moderate-to-severe	Susceptibility	22	0.988 (0.946–1.031)	0.576	0.971 (0.926–1.019)	0.233	0.969 (0.897–1.046)	0.424	0.907 (0.747–1.100)	0.333
asthma	Hospitalization	22	0.967 (0.906–1.031)	0.303	0.948 (0.866–1.039)	0.252	0.939 (0.783–1.127)	0.507	1.049 (0.781–1.408)	0.755
	Severe disease	22	0.911 (0.823–1.009)	0.074	0.998 (0.870–1.145)	0.980	1.053 (0.855–1.297)	0.631	1.309 (0.832–2.06)	0.258

**TABLE 2:** Heterogeneity tests, MR-PRESSO, and MR-Egger intercept of asthma causally linked to COVID-19 susceptibility and severity.

Exposure	Outcome	Heterogeneity			MR-PRESSO	MR Egger	
		Cochrane Q	Q_df	*p*-value	P_global-value_	Intercept	*p*-value
asthma	Susceptibility	158.953	148	0.255	0.255	0.004	0.082
	Hospitalization	150.508	143	0.317	0.294	−0.006	0.248
	Severe disease	172.538	147	0.074	0.068	−0.012	0.150
moderate-to-severe asthma	Susceptibility	36.326	21	0.020	0.019	0.014	0.384
	Hospitalization	21.480	21	0.430	0.418	−0.011	0.585
	Severe disease	24.722	21	0.259	0.245	−0.052	0.124

As shown in Table 1 and Figure 1, the OR of moderate-to-severe asthma on COVID-19 susceptibility was estimated to be 0.988 (95% CI, 0.946–1.031, *p* = 0.576) using the IVW method. Similarly, non-casual association was obtained using weighted median, weighted mode and MR-Egger method (*p* = 0.233, *p* = 0.424, *p* = 0.333, respectively). The Cochran’s Q test in the IVW model suggested that there was evidence of heterogeneity although no directional pleiotropy was assessed by MR Egger intercept test across estimates of included SNPs (Table 2). MR-PRESSO suggested one horizontal pleiotropy outlier were present. After removing the outlier (rs3997872), no heterogeneity for moderate-to-severe asthma remained (*p* = 0.244) and consistent null causal association were observed (OR = 1.001, 95% CI, 0.965–1.039, *p* = 0.952 using the IVW method). Furthermore, no significant causal effects of the genetic instrument modeling moderate-to-severe asthma on COVID-19 hospitalization and severe disease were observed, which were consistent across the 4 MR methods (all *p* > 0.05, Table 1). For both outcomes, we identified no heterogeneity of effects (*p* = 0.430; *p* = 0.259) or MR-PRESSO (*p* = 0.418; *p* = 0.245) or MR Egger intercept (*p* = 0.585; *p* = 0.124) or outlying genetic variants by the leave-one-out analysis (Table 2 and Supplementary Figures S3, S5, S6).

## Discussion

In this two-sample MR study, adopting largest possible and well-powered GWAS studies, we demonstrated no evidence showing the causal effect of asthma and moderate-to-severe asthma on COVID-19 susceptibility or severity. The results from alternative MR methods were overall robust to sensitivity analyses accounting for horizontal pleiotropy.

Consistent with our findings, multiple published traditional observational studies have not proven that asthma is harmful (Broadhurst et al., 2020; Chhiba et al., 2020; Grasselli et al., 2020; Guan et al., 2020; Epidemiology Working Group for NCIP Epidemic Response, Chinese Center for Disease Control and PreventionThe Novel Coronavirus Pneumonia Emergency Response Epidemiology Team, 2020; Wu and McGoogan, 2020; Zhang et al., 2020; Wu et al., 2021). For instance, a study reported on 1,590 patients in China, among whom no patients had physician-diagnosed asthma (Guan et al., 2020). Another study from Lombardy, Italy has also shown that the prevalence of asthma is relatively low (Grasselli et al., 2020). Asthma is absent from the top 10 comorbidities according to the fatality statistics of the New York State (New York State Department of Health COVID-19 Tracker-Fatality, 2020). In addition, a recent review of data in adults revealed that asthma appear to be under-represented in the comorbidities reported for patients with COVID-19, compared with global estimates of prevalence for these conditions in the general population (Halpin et al., 2020). Furthermore, a meta-analysis of 131 studies (410 382 patients) reported that no significant difference in asthma prevalence was found between hospitalized and non-hospitalized [RR (risk ratio) = 1.15, 95% CI, 0.92–1.43], severe and non-severe (RR = 1.21, 95% CI, 0.92–1.57) patients with COVID-19 (Liu et al., 2021), which was in line with findings from the present study. More compellingly, the study using large population-based prospective cohort demonstrated the absence of association between the existing genetic polygenic score for asthma and COVID-19 (Zhu et al., 2020). Taken together, the above findings collectively suggest that asthma may not be a predisposing or aggravating factor for COVID-19.

The theoretical mechanisms may include the role of type 2 immune response, including type 2 cytokines and accumulation of eosinophils. Some type 2 cytokines (IL-4, IL-9, IL-13, etc.) have inhibitory effects on the production of proinflammatory cytokines (IL-1β, IL-6, TNF-α, etc.), the overactivation of which have been proposed as a potential key mechanism of protection against COVID-19 to some extent (Liu et al., 2020). Local eosinophilia, which is a characteristic of asthma, was proved to be one protective mechanisms from virus infection in a mouse model (Sabogal Piñeros et al., 2019) and against severe COVID-19 illness (Ferastraoaru et al., 2021). Whereas there is lack of concrete explanation, clinical manifestations of COVID-19 may depend on the distribution of the angiotensin converting enzyme 2 (ACE2) in the respiratory airway epithelium (Wu et al., 2021). ACE2 is a transmembrane endopeptidase that cleaves both angiotensin 1 and 2 (20), and was proved to be the entry receptor for SARS-CoV-2 associated with *in vitro* susceptibility (Kimura et al., 2020). Furthermore, cofactors facilitating SARS-CoV-2 infectivity are the transmembrane peptidase serine 2 (TMPRSS2), a clinically proven inhibitor of the cellular serine protease that modifies spike proteins in multiple virus to promote viral infection and spread, and which can block the host cell entry of SARS-CoV-2, and possibly the protease furin (Kimura et al., 2020; Ferastraoaru et al., 2021). Consistent with our findings, a study reported that there were no differences in ACE2, TMPRESS2, and furin epithelial and airway gene expression between healthy volunteers and patients with asthma across all treatment intensities and severity (Bradding et al., 2020). Meanwhile, Kimura et al. (2020) demonstrated that IL-13, a type 2 cytokine associated with asthma, which significantly suppressed ACE2 and increased TMPRESS 2 expression *ex vivo* in airway epithelial cells.

A major strength of current study is the MR design. The causal relationship between two diseases can be accessed by MR analysis, however, this is not feasible in an RCT, because it is not ethical to keep the patients with one disease untreated in order to observe the occurrence and severity of another disease outcome (Li et al., 2020). Specifically, MR approach can mitigate residual confounding and reverse causality through the use of genetic variants as proxies which are fixed at conception. Another important strength is the application of the most recent and largest database for asthma and latest GWAS meta-analysis for COVID-19, which provided a possibility to assess the association between asthma and COVID-19. Nonetheless, our MR study still exist multiple limitations. First, our results may not be applicable to be extrapolated to the populations outside Europe, because the ancestry of participants included in this study was restricted to European populations. Whereas this may also reduce the bias caused by population stratification. Second, the use of a MR framework precluded directly examining the role of asthma and moderate-to-severe asthma do not have robust genetic variants available to serve as proxies (e.g., age, inhaled corticosteroid). Lastly, like all MR studies, horizontal pleiotropy is the common limitation. To test this bias, a range of sensitivity analyses were conducted. Hence, whereas the residual pleiotropy might remain, we still believe it unlikely to change the conclusions of this study.

## Conclusion

In summary, we demonstrate, for the first time leveraging a two-sample MR approach with an adequate statistical power, that genetically predicted asthma and moderate-to-severe asthma were not associated with COVID-19 susceptibility and severity, suggesting that asthma is unlikely to be a causal factor in the development of COVID-19.

## Data Availability

Publicly available datasets were analyzed in this study. Asthma genetic data are available here: https://www.ebi.ac.uk/gwas/publications/31619474. COVID-19 genetic data are available here: https://www.covid19hg.org/results/r5/.

## Supplementary Material

Supplementary Figure 1 Study design of the current study: Mendelian randomization analysis. The MR approach relies on the following three assumptions: assumption 1, the genetic variants must be powerfully correlated with the exposure (here, asthma); assumption 2, the genetic variants must be unrelated to any confounding factors that are associated with the outcome (here, COVID-19 susceptibility and severity); assumption 3, the genetic variants must affect the outcome only through exposure factors rather than via alternative ways (also known as an absence of horizontal pleiotropy).

Supplemental Figure S2 Forest plot individual and combined SNP MR-estimated effects sizes for asthma on COVID-19 susceptibility, hospitalization and severe disease.

Supplemental Figure S3 Funnel plots to show symmetrical distribution of individual variant estimates around the point estimate. **(A-C)** Funnel plots for asthma and **(D-F)** Funnel plots for moderate-to-severe asthma. The x-axis represents the MR estimate of individual variants; the y-axis represents the inverse of their standard error. Effects were estimated for asthma and moderate-to-severe asthma on COVID-19 susceptibility, hospitalization and severe disease, respective.

Supplemental Figure S4 Leave-one-out analysis related to the asthma genetic instrument with respect to COVID-19 susceptibility, hospitalization and severe disease.

Supplemental Figure S5 Forest plot individual and combined SNP MR-estimated effects sizes for moderate-to-severe asthma on COVID-19 susceptibility, hospitalization and severe disease.

Supplemental Figure S6 Leave-one-out analysis related to the moderate-to-severe asthma genetic instrument with respect to COVID-19 susceptibility, hospitalization and severe disease.

Supplemental Table S1 Sources of data for the analysis.

Supplemental Table S2 Detailed information for instrumental variables of asthma and moderate-to-severe asthma and association with COVID-19 susceptibility, hospitalization and severe disease.

Supplemental Table S3 Power calculation for the association estimates in Mendelian randomization analysis.
