# Supplementary figures and images for: Non-Causal Effects of Asthma on COVID-19 Susceptibility and Severity
Source: Front Genet. 2022 Jan 10;12:762697. doi: 10.3389/fgene.2021.762697 (PMC8784851; doi:10.3389/fgene.2021.762697)

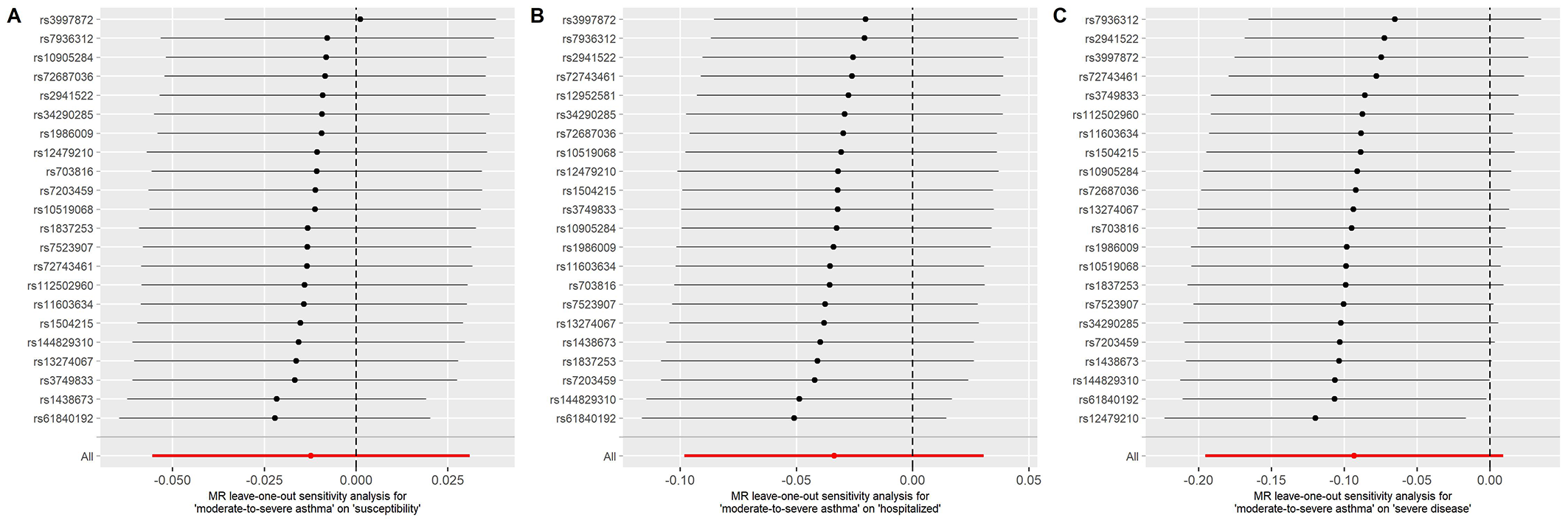

Supplement: Supplementary file 1 [file Image6.tif]

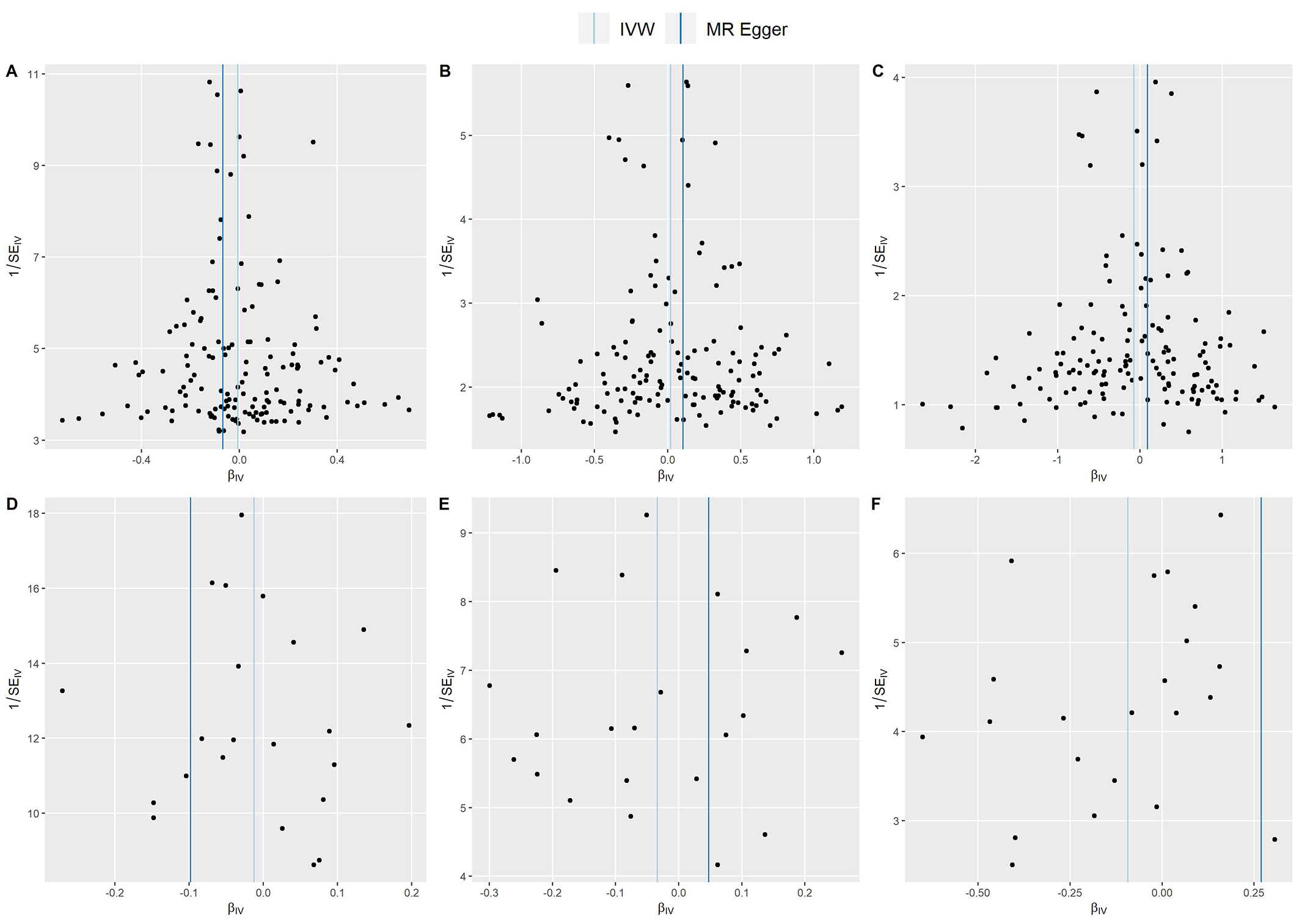

Supplement: Supplementary file 3 [file Image3.tif]

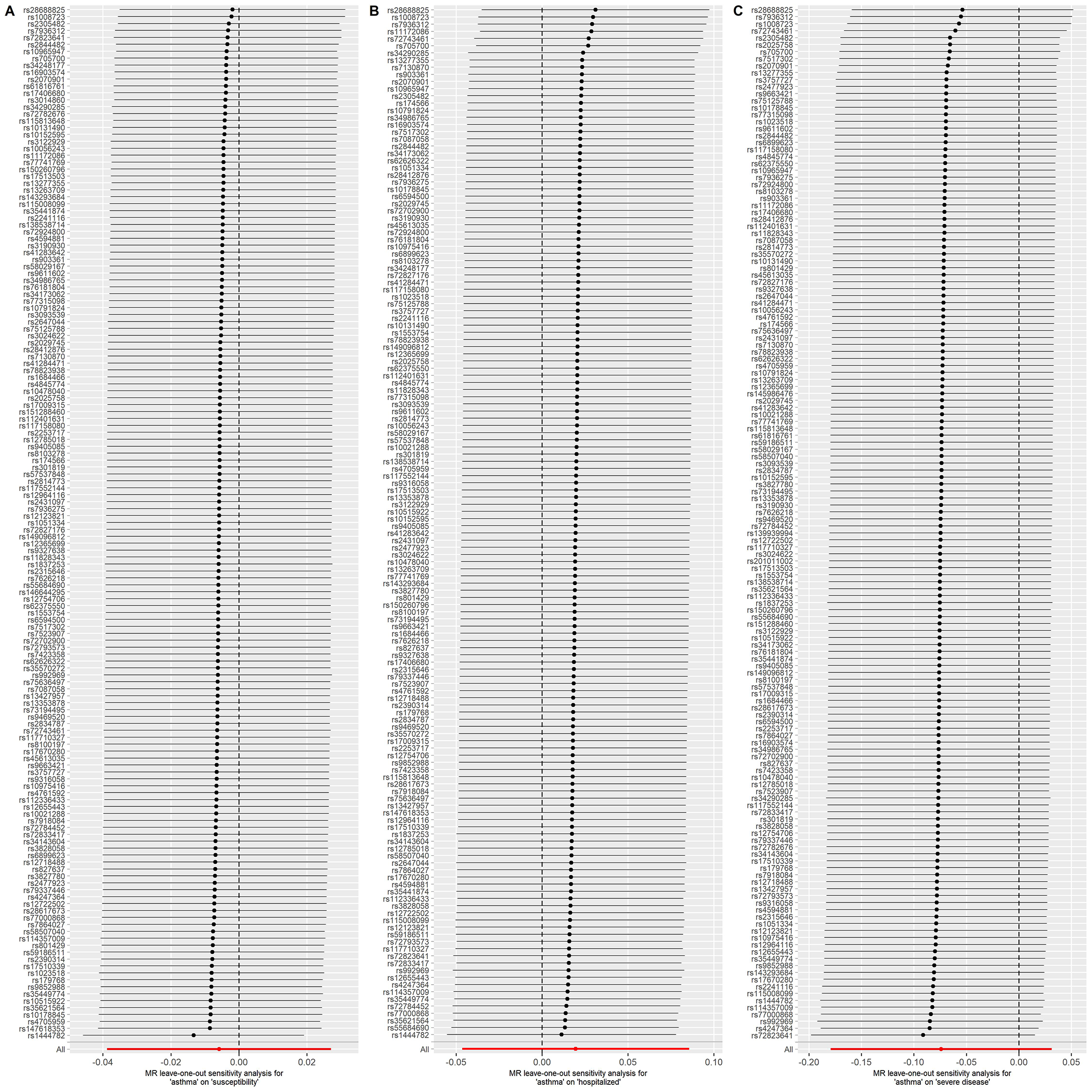

Supplement: Supplementary file 4 [file Image4.tif]

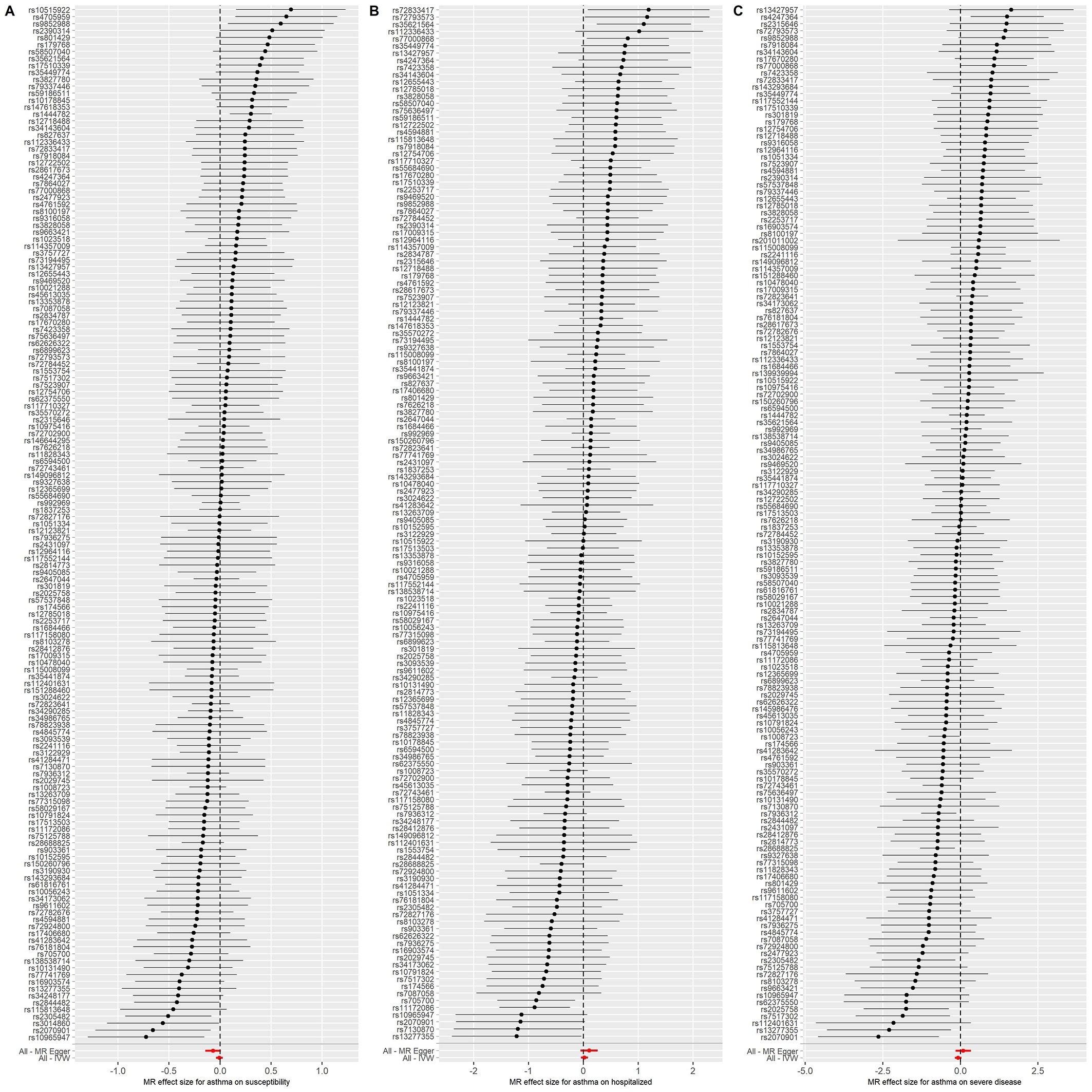

Supplement: Supplementary file 5 [file Image2.tif]

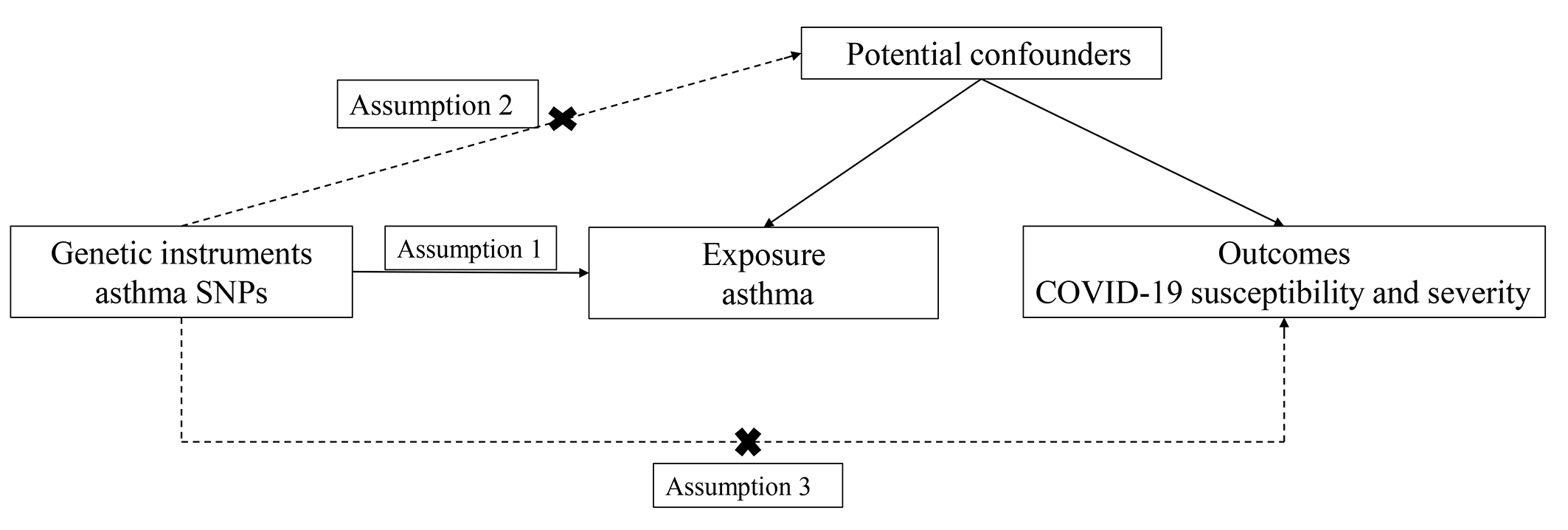

Supplement: Supplementary file 6 [file Image1.tif]

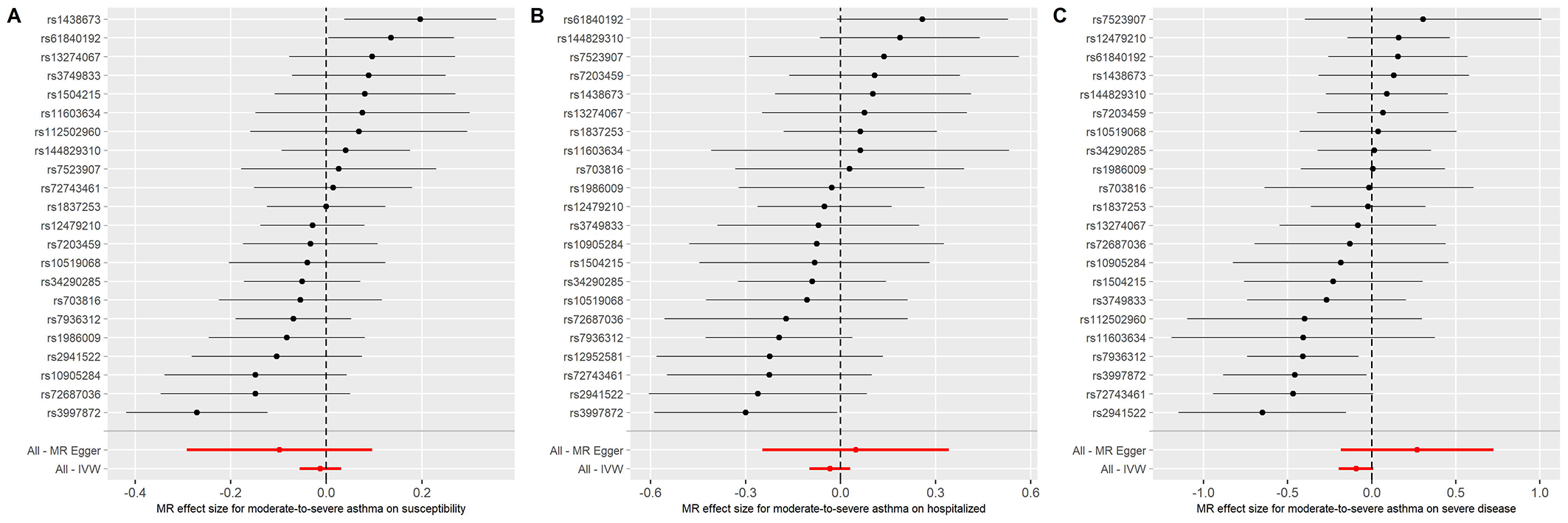

Supplement: Supplementary file 9 [file Image5.tif]
