# Supplementary material for: Non-Causal Effects of Asthma on COVID-19 Susceptibility and Severity
Source: Front Genet. 2022 Jan 10;12:762697. doi: 10.3389/fgene.2021.762697 (PMC8784851; doi:10.3389/fgene.2021.762697)
Supplement: Supplementary file 2 [file Table1.docx]

**Supplementary Table 1.** Sources of data for the analysis

| **Phenotype** | **Source of genetic variants** | | |
| --- | --- | --- | --- |
|  | **Consortium** | **Population** | **Participants** |
| Asthma |  |  |  |
| Asthma | UK Biobank [1] | European | Cases: 46 802 asthma cases without chronic obstructive pulmonary disease, emphysema, or chronic bronchitis (self-reports or ICD-10 codes) |
|  |  |  | Controls: 347 481 controls without chronic obstructive pulmonary disease, emphysema, or chronic bronchitis (self-reports or ICD-10 codes) |
| Moderate-to-severe asthma | GASP initiative, U-BIOPRED project, and UK Biobank [2] | European | Cases: 10 549 moderate-to-severe asthma cases who were taking appropriate medication or had been diagnosed by a doctor |
|  |  |  | Controls: 47 146 controls not having asthma, rhinitis, eczema, allergy, emphysema, or chronic bronchitis as diagnosed by a doctor |
| COVID-19 |  |  |  |
| Susceptibility | COVID-19 HGI [3] | European | Cases: 38 984 individuals with confirmed COVID-19 |
|  |  |  | Controls: 1 644 784 general population |
| Severity |  |  |  |
| Hospitalized | COVID-19 HGI [3] | European | Cases: 9 986 hospitalized COVID-19 patients |
|  |  |  | Controls: 1 877 672 general population |
| Severe disease | COVID-19 HGI [3] | European | Cases: 5 101 COVID-19-infected individuals who had very severe respiratory |
|  |  |  | Controls: 1 383 241 general population |

Summary statistics of SNPs were retrived from the largest availabael genome wide association studies (GWASs) for asthma [1], moderate-to-severe asthma [2], and COVID-19 susceptibility, hospitalized, and severe disease [3]. GASP, the Genetics of Asthma Severity and Phenotypes; U-BIOPRED, the Unbiased BIOmarkers in PREDiction of respiratory disease outcommes; COVID-19 HGI, COVID-19 Host Genetics Initiative.

REFERENCE

[1] Zhu, Z., Zhu, X., Liu, C.-L., Shi, H., Shen, S., Yang, Y., et al. (2019). Shared Genetics of Asthma and Mental Health Disorders: a Large-Scale Genome-wide CrossTrait Analysis. *Eur. Respir. J*. 54, 1901507. doi:10.1183/13993003.01507-2019

[2] Shrine, N., Portelli, M. A., John, C., Soler Artigas, M., Bennett, N., Hall, R., et al. (2019). Moderate-to-severe Asthma in Individuals of European Ancestry: a Genome-wide Association Study. *Lancet Respir. Med*. 7, 20-34. doi:10.1016/S2213-2600(18)30389-8

[3] The COVID-19 Host Genetics Initiative (2020). The COVID-19 Host Genetics Initiative, a Global Initiative to Elucidate the Role of Host Genetic Factors in Susceptibility and Severity of the SARS-CoV-2 Virus Pandemic. *Eur. J. Hum. Genet*. 28, 715-718. doi:10.1038/s41431-020-0636-6
