# Supplementary material for: Non-Causal Effects of Asthma on COVID-19 Susceptibility and Severity
Source: Front Genet. 2022 Jan 10;12:762697. doi: 10.3389/fgene.2021.762697 (PMC8784851; doi:10.3389/fgene.2021.762697)
Supplement: Supplementary file 7 [file Table2.docx]

**Supplementary Table 2.** Detailed information for instrumental variables of asthma and moderate-to-severe asthma and association with COVID-19

| **Chr** | **Position** | **rsID** | **EA/**  **OA** | **EAF** | **beta** | **SE** | **P-value** | **R^2^** | **F-statistic** | **Association with susceptibility** | | | **Association with hospitalization** | | | **Association with severe disease** | | |
| --- | --- | --- | --- | --- | --- | --- | --- | --- | --- | --- | --- | --- | --- | --- | --- | --- | --- | --- |
|  |  |  |  |  |  |  |  |  |  | **beta** | **SE** | **P-value** | **beta** | **SE** | **P-value** | **beta** | **SE** | **P-value** |
| **Instrumental variables of asthma** | | | |  |  |  |  |  |  |  |  |  |  |  |  |  |  |  |
| 4 | 123005534 | rs10021288 | G/A | 0.756 | -0.056 | 0.007 | 4.40E-15 | 1.62E-04 | 64 | -0.007 | 0.011 | 5.43E-01 | 0.003 | 0.021 | 8.90E-01 | 0.01 | 0.031 | 7.40E-01 |
| 5 | 110259077 | rs10056243 | G/A | 0.934 | -0.087 | 0.012 | 2.60E-12 | 1.33E-04 | 53 | 0.019 | 0.018 | 2.96E-01 | 0.01 | 0.038 | 7.95E-01 | 0.044 | 0.062 | 4.83E-01 |
| 17 | 38066267 | rs1008723 | G/T | 0.489 | 0.102 | 0.006 | 1.50E-62 | 7.32E-04 | 289 | -0.012 | 0.009 | 1.90E-01 | -0.028 | 0.018 | 1.32E-01 | -0.054 | 0.026 | 4.26E-02 |
| 14 | 68743307 | rs10131490 | A/G | 0.72 | -0.048 | 0.007 | 3.20E-12 | 1.19E-04 | 47 | 0.015 | 0.011 | 1.59E-01 | 0.009 | 0.022 | 6.82E-01 | 0.031 | 0.036 | 3.87E-01 |
| 15 | 67475488 | rs10152595 | C/G | 0.751 | 0.065 | 0.007 | 9.90E-20 | 2.19E-04 | 86 | -0.012 | 0.011 | 2.79E-01 | 0.002 | 0.024 | 9.49E-01 | -0.008 | 0.039 | 8.32E-01 |
| 2 | 8443803 | rs10178845 | G/A | 0.705 | 0.057 | 0.007 | 2.90E-17 | 1.68E-04 | 66 | 0.018 | 0.011 | 8.59E-02 | -0.014 | 0.021 | 5.02E-01 | -0.034 | 0.03 | 2.54E-01 |
| 5 | 131793772 | rs1023518 | G/T | 0.736 | -0.074 | 0.007 | 1.50E-25 | 2.83E-04 | 112 | -0.012 | 0.011 | 2.51E-01 | 0.006 | 0.021 | 7.83E-01 | 0.03 | 0.031 | 3.38E-01 |
| 5 | 110590399 | rs10478040 | C/T | 0.949 | 0.086 | 0.014 | 3.60E-10 | 9.57E-05 | 38 | -0.006 | 0.021 | 7.62E-01 | 0.008 | 0.041 | 8.54E-01 | 0.036 | 0.061 | 5.59E-01 |
| 12 | 71523134 | rs1051334 | A/C | 0.591 | 0.04 | 0.006 | 1.60E-10 | 1.13E-04 | 44 | 0 | 0.01 | 9.82E-01 | -0.018 | 0.019 | 3.44E-01 | 0.031 | 0.027 | 2.54E-01 |
| 2 | 102914654 | rs10515922 | A/G | 0.903 | -0.059 | 0.011 | 7.90E-09 | 7.30E-05 | 29 | -0.041 | 0.016 | 1.10E-02 | 0 | 0.032 | 9.99E-01 | -0.017 | 0.047 | 7.25E-01 |
| 11 | 65559266 | rs10791824 | A/G | 0.434 | -0.04 | 0.006 | 4.30E-11 | 1.13E-04 | 44 | 0.006 | 0.01 | 5.26E-01 | 0.027 | 0.021 | 1.82E-01 | 0.019 | 0.034 | 5.85E-01 |
| 9 | 23588583 | rs10965947 | C/T | 0.542 | 0.034 | 0.006 | 3.30E-08 | 8.14E-05 | 32 | -0.025 | 0.01 | 1.30E-02 | -0.038 | 0.021 | 6.54E-02 | -0.059 | 0.035 | 9.21E-02 |
| 9 | 6051924 | rs10975416 | T/G | 0.813 | 0.099 | 0.008 | 4.80E-36 | 3.88E-04 | 153 | 0.004 | 0.013 | 7.59E-01 | -0.009 | 0.026 | 7.43E-01 | 0.027 | 0.041 | 5.03E-01 |
| 12 | 57449206 | rs11172086 | C/T | 0.911 | 0.094 | 0.011 | 8.00E-18 | 1.85E-04 | 73 | -0.015 | 0.017 | 3.71E-01 | -0.084 | 0.031 | 6.77E-03 | -0.034 | 0.044 | 4.34E-01 |
| 3 | 196349004 | rs112336433 | C/T | 0.933 | 0.067 | 0.012 | 3.20E-08 | 7.91E-05 | 31 | 0.016 | 0.02 | 4.09E-01 | 0.068 | 0.04 | 8.60E-02 | 0.02 | 0.059 | 7.29E-01 |
| 17 | 38764524 | rs112401631 | T/A | 0.977 | -0.138 | 0.022 | 1.50E-10 | 9.98E-05 | 39 | 0.011 | 0.043 | 7.92E-01 | 0.049 | 0.094 | 6.01E-01 | 0.297 | 0.176 | 9.06E-02 |
| 6 | 28965512 | rs114357009 | G/A | 0.95 | -0.147 | 0.023 | 1.60E-10 | 1.04E-04 | 41 | -0.023 | 0.023 | 3.08E-01 | -0.057 | 0.043 | 1.84E-01 | -0.074 | 0.061 | 2.21E-01 |
| 5 | 131991881 | rs115008099 | C/T | 0.832 | -0.098 | 0.008 | 6.20E-32 | 3.80E-04 | 150 | 0.007 | 0.013 | 5.55E-01 | -0.023 | 0.026 | 3.83E-01 | -0.057 | 0.044 | 1.97E-01 |
| 1 | 152921586 | rs115813648 | T/C | 0.977 | -0.156 | 0.021 | 1.90E-13 | 1.40E-04 | 55 | 0.071 | 0.042 | 8.63E-02 | -0.091 | 0.09 | 3.13E-01 | 0.05 | 0.169 | 7.68E-01 |
| 10 | 8293923 | rs117158080 | T/C | 0.889 | 0.06 | 0.01 | 9.10E-10 | 9.13E-05 | 36 | -0.004 | 0.016 | 8.21E-01 | -0.018 | 0.03 | 5.64E-01 | -0.058 | 0.044 | 1.88E-01 |
| 19 | 3136091 | rs117552144 | C/T | 0.933 | -0.084 | 0.013 | 9.70E-11 | 1.06E-04 | 42 | 0.002 | 0.023 | 9.42E-01 | 0.005 | 0.047 | 9.10E-01 | -0.079 | 0.08 | 3.22E-01 |
| 19 | 33726578 | rs117710327 | C/A | 0.933 | 0.12 | 0.013 | 1.20E-21 | 2.16E-04 | 85 | 0.006 | 0.02 | 7.50E-01 | 0.06 | 0.044 | 1.76E-01 | 0.007 | 0.073 | 9.21E-01 |
| 11 | 111491322 | rs11828343 | A/G | 0.717 | 0.037 | 0.007 | 2.00E-08 | 7.09E-05 | 28 | 0.001 | 0.01 | 9.32E-01 | -0.008 | 0.02 | 6.99E-01 | -0.03 | 0.028 | 2.88E-01 |
| 1 | 152179152 | rs12123821 | C/T | 0.952 | -0.152 | 0.014 | 1.20E-25 | 2.99E-04 | 118 | 0.001 | 0.024 | 9.71E-01 | -0.051 | 0.047 | 2.84E-01 | -0.052 | 0.07 | 4.59E-01 |
| 11 | 118743286 | rs12365699 | G/A | 0.833 | 0.055 | 0.008 | 8.90E-09 | 1.20E-04 | 47 | 0.001 | 0.013 | 9.59E-01 | -0.01 | 0.027 | 6.98E-01 | -0.023 | 0.046 | 6.24E-01 |
| 5 | 141467856 | rs12655443 | C/A | 0.496 | -0.047 | 0.006 | 2.20E-14 | 1.56E-04 | 61 | -0.006 | 0.01 | 5.40E-01 | -0.03 | 0.019 | 1.12E-01 | -0.032 | 0.026 | 2.29E-01 |
| 11 | 76366690 | rs12718488 | T/G | 0.653 | -0.038 | 0.006 | 9.30E-09 | 1.02E-04 | 40 | -0.011 | 0.01 | 2.77E-01 | -0.014 | 0.019 | 4.81E-01 | -0.031 | 0.028 | 2.67E-01 |
| 10 | 6093139 | rs12722502 | C/T | 0.982 | 0.195 | 0.023 | 6.30E-17 | 1.82E-04 | 72 | 0.047 | 0.042 | 2.66E-01 | 0.116 | 0.086 | 1.77E-01 | 0.005 | 0.123 | 9.69E-01 |
| 1 | 151590206 | rs12754706 | A/G | 0.964 | -0.095 | 0.017 | 1.70E-08 | 7.92E-05 | 31 | -0.005 | 0.027 | 8.40E-01 | -0.051 | 0.054 | 3.48E-01 | -0.079 | 0.082 | 3.34E-01 |
| 10 | 8515348 | rs12785018 | C/T | 0.661 | -0.042 | 0.007 | 1.10E-10 | 9.13E-05 | 36 | 0.002 | 0.011 | 8.46E-01 | -0.027 | 0.022 | 2.25E-01 | -0.028 | 0.036 | 4.37E-01 |
| 18 | 61442619 | rs12964116 | A/G | 0.965 | -0.098 | 0.017 | 2.30E-09 | 8.43E-05 | 33 | 0.001 | 0.025 | 9.53E-01 | -0.043 | 0.045 | 3.41E-01 | -0.078 | 0.063 | 2.16E-01 |
| 8 | 81287175 | rs13263709 | T/C | 0.351 | 0.066 | 0.007 | 1.90E-24 | 2.25E-04 | 89 | -0.008 | 0.011 | 4.37E-01 | 0.003 | 0.021 | 8.75E-01 | -0.014 | 0.035 | 6.84E-01 |
| 8 | 128777719 | rs13277355 | A/G | 0.274 | 0.038 | 0.007 | 1.80E-08 | 7.47E-05 | 29 | -0.015 | 0.011 | 1.61E-01 | -0.046 | 0.023 | 4.34E-02 | -0.087 | 0.039 | 2.43E-02 |
| 5 | 156960581 | rs13353878 | G/A | 0.84 | -0.05 | 0.008 | 1.40E-09 | 9.91E-05 | 39 | -0.006 | 0.013 | 6.61E-01 | 0.002 | 0.025 | 9.37E-01 | 0.006 | 0.035 | 8.67E-01 |
| 2 | 102689031 | rs13427957 | C/T | 0.574 | -0.035 | 0.006 | 2.20E-08 | 8.63E-05 | 34 | -0.005 | 0.01 | 6.51E-01 | -0.026 | 0.022 | 2.25E-01 | -0.057 | 0.036 | 1.10E-01 |
| 4 | 123115272 | rs138538714 | C/T | 0.972 | 0.107 | 0.019 | 1.70E-08 | 8.04E-05 | 32 | -0.032 | 0.029 | 2.63E-01 | -0.007 | 0.056 | 8.99E-01 | 0.017 | 0.076 | 8.25E-01 |
| 9 | 6380605 | rs139939994 | C/G | 0.991 | -0.193 | 0.034 | 1.2E-08 | 8.17E-05 | 32 | - | - | - | - | - | - | -0.056 | 0.236 | 8.14E-01 |
| 9 | 5924109 | rs143293684 | A/T | 0.979 | -0.164 | 0.022 | 2.00E-14 | 1.41E-04 | 56 | 0.035 | 0.036 | 3.29E-01 | -0.016 | 0.072 | 8.30E-01 | -0.162 | 0.102 | 1.15E-01 |
| 10 | 9058671 | rs1444782 | G/A | 0.576 | 0.092 | 0.006 | 1.10E-48 | 5.96E-04 | 235 | 0.028 | 0.01 | 4.08E-03 | 0.03 | 0.019 | 1.09E-01 | 0.019 | 0.027 | 4.78E-01 |
| 16 | 27359515 | rs145986476 | C/A | 0.993 | 0.269 | 0.038 | 1.2E-12 | 1.27E-04 | 50 | - | - | - | - | - | - | -0.121 | 0.245 | 6.20E-01 |
| 17 | 37574592 | rs146644295 | G/C | 0.978 | -0.164 | 0.022 | 1.80E-14 | 1.41E-04 | 56 | -0.005 | 0.035 | 8.94E-01 | - | - | - | - | - | - |
| 16 | 27344694 | rs147618353 | C/T | 0.993 | 0.269 | 0.038 | 1.20E-12 | 1.27E-04 | 50 | 0.084 | 0.047 | 7.59E-02 | 0.085 | 0.106 | 4.20E-01 | - | - | - |
| 5 | 110418241 | rs149096812 | T/C | 0.979 | 0.119 | 0.022 | 2.00E-08 | 7.42E-05 | 29 | 0.002 | 0.037 | 9.55E-01 | -0.042 | 0.075 | 5.80E-01 | 0.061 | 0.106 | 5.65E-01 |
| 17 | 38168828 | rs150260796 | A/G | 0.982 | -0.169 | 0.024 | 1.90E-12 | 1.26E-04 | 50 | 0.033 | 0.033 | 3.28E-01 | -0.023 | 0.078 | 7.71E-01 | -0.04 | 0.132 | 7.61E-01 |
| 1 | 151587185 | rs151288460 | T/G | 0.966 | -0.106 | 0.018 | 6.60E-09 | 8.79E-05 | 35 | 0.009 | 0.033 | 7.88E-01 | - | - | - | -0.049 | 0.104 | 6.40E-01 |
| 17 | 46563707 | rs1553754 | G/T | 0.44 | -0.035 | 0.006 | 2.00E-08 | 8.63E-05 | 34 | -0.003 | 0.01 | 8.00E-01 | 0.012 | 0.021 | 5.61E-01 | -0.011 | 0.034 | 7.39E-01 |
| 3 | 196359310 | rs1684466 | G/A | 0.362 | 0.052 | 0.007 | 3.90E-15 | 1.40E-04 | 55 | -0.003 | 0.011 | 7.81E-01 | 0.007 | 0.022 | 7.43E-01 | 0.015 | 0.035 | 6.67E-01 |
| 5 | 14610309 | rs16903574 | C/G | 0.924 | -0.083 | 0.012 | 3.80E-12 | 1.21E-04 | 48 | 0.033 | 0.018 | 7.57E-02 | 0.052 | 0.041 | 2.01E-01 | -0.053 | 0.072 | 4.62E-01 |
| 2 | 30491392 | rs17009315 | A/G | 0.768 | -0.042 | 0.007 | 4.90E-09 | 9.13E-05 | 36 | 0.003 | 0.011 | 7.82E-01 | -0.019 | 0.022 | 4.07E-01 | -0.017 | 0.033 | 6.02E-01 |
| 10 | 9208204 | rs17406680 | G/C | 0.948 | -0.125 | 0.014 | 6.40E-20 | 2.02E-04 | 80 | 0.032 | 0.023 | 1.58E-01 | -0.023 | 0.051 | 6.54E-01 | 0.107 | 0.096 | 2.66E-01 |
| 11 | 61592362 | rs174566 | A/G | 0.649 | 0.037 | 0.006 | 4.80E-09 | 9.64E-05 | 38 | -0.002 | 0.01 | 8.62E-01 | -0.028 | 0.019 | 1.54E-01 | -0.02 | 0.028 | 4.84E-01 |
| 5 | 132029414 | rs17510339 | C/T | 0.876 | -0.064 | 0.009 | 1.70E-11 | 1.28E-04 | 51 | -0.025 | 0.014 | 7.57E-02 | -0.031 | 0.031 | 3.08E-01 | -0.059 | 0.054 | 2.74E-01 |
| 5 | 110146446 | rs17513503 | C/G | 0.926 | -0.099 | 0.012 | 3.70E-17 | 1.73E-04 | 68 | 0.015 | 0.018 | 3.78E-01 | 0.001 | 0.033 | 9.80E-01 | -0.002 | 0.048 | 9.74E-01 |
| 3 | 188400239 | rs17670280 | G/C | 0.832 | 0.057 | 0.008 | 4.90E-12 | 1.29E-04 | 51 | 0.006 | 0.013 | 6.27E-01 | 0.028 | 0.025 | 2.57E-01 | 0.063 | 0.037 | 9.14E-02 |
| 16 | 27424171 | rs179768 | T/C | 0.533 | -0.042 | 0.006 | 1.80E-11 | 1.24E-04 | 49 | -0.02 | 0.01 | 4.79E-02 | -0.015 | 0.021 | 4.84E-01 | -0.036 | 0.034 | 2.89E-01 |
| 5 | 110401872 | rs1837253 | T/C | 0.261 | -0.106 | 0.007 | 1.20E-51 | 5.81E-04 | 229 | 0 | 0.011 | 9.97E-01 | -0.011 | 0.021 | 6.15E-01 | 0.004 | 0.03 | 9.02E-01 |
| 12 | 57498220 | rs201011002 | T/C | 0.995 | 0.248 | 0.044 | 1.5E-08 | 8.06E-05 | 32 | - | - | - | - | - | - | 0.147 | 0.33 | 6.55E-01 |
| 10 | 8841669 | rs2025758 | T/C | 0.543 | 0.049 | 0.006 | 3.90E-15 | 1.69E-04 | 67 | -0.002 | 0.01 | 8.25E-01 | -0.006 | 0.021 | 7.54E-01 | -0.086 | 0.034 | 1.26E-02 |
| 19 | 6580892 | rs2029745 | T/C | 0.609 | -0.036 | 0.006 | 2.10E-08 | 9.13E-05 | 36 | 0.004 | 0.01 | 6.65E-01 | 0.023 | 0.021 | 2.65E-01 | 0.016 | 0.034 | 6.45E-01 |
| 1 | 161185058 | rs2070901 | G/T | 0.73 | -0.039 | 0.007 | 1.80E-08 | 7.87E-05 | 31 | 0.026 | 0.011 | 2.25E-02 | 0.045 | 0.023 | 5.57E-02 | 0.103 | 0.039 | 7.94E-03 |
| 2 | 103003265 | rs2241116 | C/A | 0.78 | -0.072 | 0.007 | 1.40E-22 | 2.68E-04 | 106 | 0.008 | 0.011 | 4.95E-01 | 0.006 | 0.022 | 7.89E-01 | -0.041 | 0.033 | 2.10E-01 |
| 17 | 38868236 | rs2253717 | G/A | 0.715 | 0.042 | 0.007 | 3.40E-10 | 9.13E-05 | 36 | -0.002 | 0.011 | 8.40E-01 | 0.02 | 0.023 | 3.82E-01 | 0.028 | 0.038 | 4.64E-01 |
| 17 | 38140927 | rs2305482 | A/C | 0.457 | 0.044 | 0.006 | 6.30E-13 | 1.36E-04 | 54 | -0.022 | 0.01 | 1.86E-02 | -0.021 | 0.018 | 2.48E-01 | -0.059 | 0.027 | 2.62E-02 |
| 20 | 62379750 | rs2315646 | T/G | 0.659 | -0.038 | 0.007 | 2.90E-09 | 7.47E-05 | 29 | -0.002 | 0.011 | 8.82E-01 | -0.014 | 0.022 | 5.35E-01 | -0.056 | 0.035 | 1.12E-01 |
| 7 | 20455978 | rs2390314 | A/T | 0.07 | 0.072 | 0.012 | 2.80E-09 | 9.13E-05 | 36 | 0.037 | 0.019 | 5.06E-02 | 0.031 | 0.04 | 4.36E-01 | 0.051 | 0.069 | 4.58E-01 |
| 5 | 159890885 | rs2431097 | C/T | 0.515 | -0.035 | 0.006 | 2.50E-08 | 8.63E-05 | 34 | 0.001 | 0.01 | 9.59E-01 | -0.004 | 0.021 | 8.59E-01 | 0.025 | 0.034 | 4.68E-01 |
| 10 | 8565990 | rs2477923 | T/C | 0.537 | 0.045 | 0.006 | 5.60E-13 | 1.43E-04 | 56 | 0.01 | 0.01 | 3.19E-01 | 0.004 | 0.021 | 8.64E-01 | -0.055 | 0.034 | 1.07E-01 |
| 6 | 32667910 | rs2647044 | G/A | 0.853 | -0.142 | 0.018 | 4.40E-15 | 1.58E-04 | 62 | 0.005 | 0.016 | 7.58E-01 | -0.02 | 0.032 | 5.37E-01 | 0.03 | 0.056 | 5.87E-01 |
| 1 | 159149481 | rs2814773 | T/C | 0.732 | 0.039 | 0.007 | 1.60E-08 | 7.87E-05 | 31 | -0.001 | 0.011 | 9.25E-01 | -0.007 | 0.021 | 7.26E-01 | -0.029 | 0.03 | 3.43E-01 |
| 21 | 36502558 | rs2834787 | A/G | 0.847 | -0.056 | 0.009 | 2.40E-11 | 9.82E-05 | 39 | -0.006 | 0.014 | 6.55E-01 | -0.021 | 0.029 | 4.60E-01 | 0.011 | 0.048 | 8.21E-01 |
| 17 | 47454515 | rs28412876 | G/T | 0.624 | -0.051 | 0.006 | 2.10E-15 | 1.83E-04 | 72 | 0.003 | 0.01 | 7.48E-01 | 0.018 | 0.021 | 4.06E-01 | 0.037 | 0.036 | 3.08E-01 |
| 6 | 31539767 | rs2844482 | C/T | 0.847 | -0.062 | 0.01 | 1.40E-09 | 9.75E-05 | 38 | 0.026 | 0.013 | 4.69E-02 | 0.023 | 0.025 | 3.61E-01 | 0.044 | 0.036 | 2.28E-01 |
| 15 | 67371244 | rs28617673 | C/T | 0.89 | -0.072 | 0.01 | 4.80E-13 | 1.31E-04 | 52 | -0.017 | 0.016 | 2.67E-01 | -0.025 | 0.032 | 4.27E-01 | -0.025 | 0.052 | 6.35E-01 |
| 6 | 32587157 | rs28688825 | A/G | 0.883 | 0.129 | 0.011 | 5.10E-31 | 3.49E-04 | 138 | -0.022 | 0.014 | 1.12E-01 | -0.052 | 0.026 | 4.65E-02 | -0.096 | 0.037 | 1.02E-02 |
| 1 | 153314782 | rs3014860 | G/A | 0.039 | 0.09 | 0.016 | 2.40E-08 | 8.02E-05 | 32 | -0.051 | 0.025 | 4.58E-02 | - | - | - | - | - | - |
| 1 | 8501786 | rs301819 | A/G | 0.408 | -0.038 | 0.006 | 1.10E-09 | 1.02E-04 | 40 | 0.002 | 0.01 | 8.73E-01 | 0.005 | 0.021 | 8.20E-01 | -0.034 | 0.035 | 3.22E-01 |
| 16 | 27365453 | rs3024622 | C/G | 0.648 | -0.055 | 0.007 | 1.60E-17 | 1.57E-04 | 62 | 0.005 | 0.011 | 6.63E-01 | -0.004 | 0.023 | 8.68E-01 | -0.005 | 0.037 | 8.89E-01 |
| 6 | 31538277 | rs3093539 | G/A | 0.984 | 0.174 | 0.028 | 9.80E-10 | 9.79E-05 | 39 | -0.019 | 0.036 | 6.02E-01 | -0.025 | 0.081 | 7.58E-01 | -0.027 | 0.12 | 8.22E-01 |
| 12 | 57509102 | rs3122929 | C/T | 0.596 | -0.069 | 0.006 | 1.40E-27 | 3.35E-04 | 132 | 0.008 | 0.01 | 4.49E-01 | -0.001 | 0.021 | 9.76E-01 | -0.005 | 0.036 | 8.84E-01 |
| 6 | 128291199 | rs3190930 | C/T | 0.755 | -0.049 | 0.007 | 1.30E-11 | 1.24E-04 | 49 | 0.01 | 0.011 | 3.94E-01 | 0.021 | 0.024 | 3.80E-01 | 0.005 | 0.04 | 9.09E-01 |
| 2 | 242562010 | rs34143604 | C/A | 0.62 | -0.04 | 0.006 | 9.50E-10 | 1.13E-04 | 44 | -0.011 | 0.011 | 2.99E-01 | -0.027 | 0.022 | 2.18E-01 | -0.046 | 0.038 | 2.21E-01 |
| 8 | 145158607 | rs34173062 | G/A | 0.928 | -0.08 | 0.013 | 5.20E-10 | 9.60E-05 | 38 | 0.017 | 0.021 | 4.11E-01 | 0.052 | 0.044 | 2.30E-01 | -0.028 | 0.068 | 6.79E-01 |
| 12 | 57602632 | rs34248177 | A/G | 0.995 | 0.248 | 0.044 | 1.50E-08 | 8.06E-05 | 32 | -0.102 | 0.056 | 6.93E-02 | -0.085 | 0.126 | 5.01E-01 | - | - | - |
| 2 | 242698640 | rs34290285 | G/A | 0.744 | 0.096 | 0.007 | 4.10E-42 | 4.77E-04 | 188 | -0.009 | 0.011 | 4.18E-01 | -0.016 | 0.021 | 4.52E-01 | 0.003 | 0.03 | 9.30E-01 |
| 15 | 61069201 | rs34986765 | T/C | 0.87 | 0.083 | 0.009 | 2.00E-19 | 2.16E-04 | 85 | -0.008 | 0.014 | 5.62E-01 | -0.021 | 0.026 | 4.28E-01 | 0.011 | 0.039 | 7.80E-01 |
| 16 | 11213021 | rs35441874 | T/A | 0.753 | 0.083 | 0.007 | 1.30E-30 | 3.56E-04 | 141 | -0.007 | 0.011 | 5.54E-01 | 0.018 | 0.023 | 4.35E-01 | 0.006 | 0.039 | 8.81E-01 |
| 6 | 33031612 | rs35449774 | C/T | 0.858 | -0.069 | 0.011 | 7.30E-11 | 9.98E-05 | 39 | -0.025 | 0.014 | 7.79E-02 | -0.053 | 0.028 | 6.20E-02 | -0.067 | 0.045 | 1.36E-01 |
| 3 | 33047662 | rs35570272 | G/T | 0.604 | -0.051 | 0.006 | 3.10E-15 | 1.83E-04 | 72 | -0.002 | 0.01 | 8.18E-01 | -0.014 | 0.021 | 5.15E-01 | 0.029 | 0.034 | 3.98E-01 |
| 7 | 20586843 | rs35621564 | A/G | 0.635 | 0.049 | 0.006 | 1.20E-13 | 1.69E-04 | 67 | 0.02 | 0.01 | 5.15E-02 | 0.054 | 0.021 | 1.19E-02 | 0.01 | 0.036 | 7.91E-01 |
| 7 | 20411897 | rs3757727 | T/C | 0.598 | 0.039 | 0.006 | 2.20E-10 | 1.07E-04 | 42 | 0.006 | 0.01 | 5.30E-01 | -0.009 | 0.019 | 6.25E-01 | -0.04 | 0.027 | 1.40E-01 |
| 5 | 118690781 | rs3827780 | G/A | 0.445 | 0.034 | 0.006 | 1.60E-08 | 8.14E-05 | 32 | 0.012 | 0.01 | 2.13E-01 | 0.006 | 0.019 | 7.56E-01 | -0.005 | 0.027 | 8.52E-01 |
| 1 | 151786281 | rs3828058 | G/A | 0.626 | -0.047 | 0.006 | 1.40E-12 | 1.56E-04 | 61 | -0.008 | 0.01 | 4.10E-01 | -0.03 | 0.022 | 1.74E-01 | -0.031 | 0.037 | 3.98E-01 |
| 9 | 101915887 | rs41283642 | C/T | 0.966 | 0.108 | 0.017 | 1.80E-10 | 1.02E-04 | 40 | -0.029 | 0.03 | 3.19E-01 | 0.007 | 0.067 | 9.17E-01 | -0.059 | 0.121 | 6.26E-01 |
| 10 | 6626214 | rs41284471 | G/A | 0.799 | -0.047 | 0.008 | 5.60E-10 | 8.75E-05 | 35 | 0.005 | 0.013 | 6.92E-01 | 0.02 | 0.027 | 4.59E-01 | 0.048 | 0.048 | 3.23E-01 |
| 17 | 43336687 | rs4247364 | G/C | 0.3 | 0.047 | 0.007 | 7.60E-12 | 1.14E-04 | 45 | 0.011 | 0.01 | 2.79E-01 | 0.034 | 0.019 | 7.98E-02 | 0.07 | 0.028 | 1.19E-02 |
| 4 | 123141070 | rs45613035 | T/C | 0.901 | -0.074 | 0.01 | 1.10E-12 | 1.39E-04 | 55 | -0.009 | 0.017 | 6.11E-01 | 0.022 | 0.032 | 4.97E-01 | 0.034 | 0.047 | 4.61E-01 |
| 5 | 35846815 | rs4594881 | G/T | 0.657 | 0.042 | 0.007 | 1.00E-10 | 9.13E-05 | 36 | -0.009 | 0.01 | 3.45E-01 | 0.024 | 0.02 | 2.11E-01 | 0.03 | 0.029 | 2.89E-01 |
| 5 | 131865791 | rs4705959 | T/C | 0.743 | 0.043 | 0.007 | 2.20E-09 | 9.57E-05 | 38 | 0.028 | 0.011 | 1.06E-02 | -0.002 | 0.021 | 9.13E-01 | -0.016 | 0.03 | 6.05E-01 |
| 12 | 94595830 | rs4761592 | T/C | 0.15 | -0.049 | 0.009 | 1.40E-08 | 7.52E-05 | 30 | -0.01 | 0.014 | 4.44E-01 | -0.017 | 0.026 | 5.09E-01 | 0.027 | 0.038 | 4.73E-01 |
| 1 | 152445621 | rs4845774 | T/C | 0.428 | 0.035 | 0.006 | 1.80E-08 | 8.63E-05 | 34 | -0.004 | 0.01 | 7.23E-01 | -0.008 | 0.019 | 6.85E-01 | -0.036 | 0.027 | 1.85E-01 |
| 11 | 76057946 | rs55684690 | C/T | 0.96 | -0.176 | 0.016 | 8.30E-29 | 3.07E-04 | 121 | -0.001 | 0.026 | 9.56E-01 | -0.087 | 0.051 | 8.77E-02 | -0.003 | 0.074 | 9.68E-01 |
| 19 | 45354044 | rs57537848 | T/G | 0.483 | -0.035 | 0.006 | 2.20E-08 | 8.63E-05 | 34 | 0.002 | 0.01 | 8.74E-01 | 0.007 | 0.021 | 7.32E-01 | -0.024 | 0.034 | 4.80E-01 |
| 9 | 5850375 | rs58029167 | A/G | 0.727 | -0.056 | 0.007 | 1.10E-15 | 1.62E-04 | 64 | 0.008 | 0.011 | 4.74E-01 | 0.005 | 0.023 | 8.19E-01 | 0.01 | 0.041 | 8.12E-01 |
| 7 | 22792318 | rs58507040 | A/C | 0.713 | -0.042 | 0.007 | 1.30E-09 | 9.13E-05 | 36 | -0.019 | 0.011 | 8.93E-02 | -0.026 | 0.021 | 2.28E-01 | 0.007 | 0.031 | 8.21E-01 |
| 13 | 99986238 | rs59186511 | C/T | 0.88 | 0.069 | 0.01 | 4.40E-13 | 1.21E-04 | 48 | 0.023 | 0.015 | 1.16E-01 | 0.042 | 0.029 | 1.50E-01 | -0.01 | 0.044 | 8.13E-01 |
| 1 | 152285861 | rs61816761 | G/A | 0.977 | -0.234 | 0.022 | 5.10E-27 | 2.87E-04 | 113 | 0.05 | 0.039 | 1.95E-01 | - | - | - | 0.041 | 0.168 | 8.09E-01 |
| 5 | 110387125 | rs62375550 | G/C | 0.956 | -0.094 | 0.016 | 2.50E-09 | 8.75E-05 | 35 | -0.005 | 0.025 | 8.40E-01 | 0.024 | 0.055 | 6.58E-01 | 0.165 | 0.097 | 8.95E-02 |
| 10 | 6115639 | rs62626322 | T/G | 0.888 | -0.054 | 0.01 | 3.70E-08 | 7.40E-05 | 29 | -0.005 | 0.015 | 7.40E-01 | 0.034 | 0.029 | 2.52E-01 | 0.024 | 0.043 | 5.74E-01 |
| 5 | 110470994 | rs6594500 | G/A | 0.602 | -0.059 | 0.006 | 3.80E-20 | 2.45E-04 | 97 | -0.001 | 0.01 | 8.98E-01 | 0.014 | 0.021 | 5.00E-01 | -0.013 | 0.035 | 6.99E-01 |
| 6 | 90986559 | rs6899623 | A/G | 0.646 | 0.064 | 0.006 | 9.00E-24 | 2.88E-04 | 114 | 0.006 | 0.01 | 5.64E-01 | -0.007 | 0.019 | 7.01E-01 | -0.026 | 0.028 | 3.47E-01 |
| 12 | 56389293 | rs705700 | T/C | 0.576 | -0.052 | 0.006 | 1.10E-16 | 1.90E-04 | 75 | 0.015 | 0.01 | 1.27E-01 | 0.044 | 0.019 | 1.75E-02 | 0.05 | 0.027 | 6.18E-02 |
| 10 | 8114922 | rs7087058 | A/G | 0.828 | -0.046 | 0.008 | 1.20E-08 | 8.38E-05 | 33 | -0.005 | 0.013 | 6.89E-01 | 0.038 | 0.027 | 1.63E-01 | 0.051 | 0.044 | 2.50E-01 |
| 11 | 36344202 | rs7130870 | C/T | 0.67 | 0.038 | 0.007 | 8.40E-09 | 7.47E-05 | 29 | -0.004 | 0.011 | 6.86E-01 | -0.045 | 0.023 | 4.55E-02 | -0.025 | 0.037 | 4.97E-01 |
| 1 | 152771963 | rs72702900 | T/A | 0.962 | -0.134 | 0.016 | 5.60E-16 | 1.78E-04 | 70 | -0.005 | 0.026 | 8.53E-01 | 0.039 | 0.053 | 4.65E-01 | -0.035 | 0.08 | 6.61E-01 |
| 15 | 67441750 | rs72743461 | C/A | 0.763 | -0.102 | 0.007 | 3.60E-45 | 5.38E-04 | 212 | -0.002 | 0.011 | 8.67E-01 | 0.029 | 0.022 | 1.73E-01 | 0.061 | 0.032 | 5.44E-02 |
| 10 | 9032555 | rs72782676 | C/G | 0.993 | 0.357 | 0.038 | 6.50E-21 | 2.24E-04 | 88 | -0.08 | 0.065 | 2.16E-01 | - | - | - | 0.121 | 0.198 | 5.40E-01 |
| 10 | 9111826 | rs72784452 | C/T | 0.924 | 0.117 | 0.012 | 1.50E-23 | 2.41E-04 | 95 | 0.01 | 0.018 | 6.00E-01 | 0.052 | 0.034 | 1.30E-01 | -0.004 | 0.047 | 9.30E-01 |
| 5 | 110564273 | rs72793573 | G/A | 0.735 | -0.04 | 0.007 | 2.50E-08 | 8.28E-05 | 33 | -0.004 | 0.011 | 7.51E-01 | -0.046 | 0.023 | 4.47E-02 | -0.058 | 0.038 | 1.33E-01 |
| 2 | 102936159 | rs72823641 | T/A | 0.863 | 0.147 | 0.009 | 1.80E-60 | 6.76E-04 | 267 | -0.013 | 0.014 | 3.44E-01 | 0.019 | 0.026 | 4.69E-01 | 0.056 | 0.038 | 1.42E-01 |
| 17 | 37810795 | rs72827176 | C/T | 0.946 | 0.079 | 0.014 | 5.00E-09 | 8.08E-05 | 32 | 0 | 0.024 | 9.91E-01 | -0.042 | 0.051 | 4.10E-01 | -0.111 | 0.093 | 2.32E-01 |
| 17 | 45873049 | rs72833417 | A/T | 0.869 | -0.058 | 0.009 | 1.10E-09 | 1.05E-04 | 42 | -0.014 | 0.015 | 3.47E-01 | -0.069 | 0.033 | 3.55E-02 | -0.058 | 0.056 | 3.00E-01 |
| 11 | 65663606 | rs72924800 | G/A | 0.871 | 0.058 | 0.009 | 2.10E-10 | 1.05E-04 | 42 | -0.014 | 0.014 | 3.26E-01 | -0.024 | 0.03 | 4.27E-01 | -0.07 | 0.051 | 1.65E-01 |
| 3 | 188328476 | rs73194495 | A/T | 0.81 | -0.047 | 0.008 | 2.70E-08 | 8.75E-05 | 35 | -0.007 | 0.014 | 6.06E-01 | -0.012 | 0.031 | 6.84E-01 | 0.01 | 0.052 | 8.45E-01 |
| 2 | 228704721 | rs7423358 | T/C | 0.242 | -0.041 | 0.007 | 9.60E-09 | 8.70E-05 | 34 | -0.004 | 0.012 | 7.29E-01 | -0.029 | 0.027 | 2.78E-01 | -0.042 | 0.044 | 3.36E-01 |
| 10 | 9255890 | rs75125788 | C/T | 0.919 | 0.062 | 0.011 | 4.90E-08 | 8.06E-05 | 32 | -0.01 | 0.017 | 5.46E-01 | -0.02 | 0.034 | 5.60E-01 | -0.084 | 0.05 | 9.42E-02 |
| 1 | 25254317 | rs7517302 | C/T | 0.423 | -0.034 | 0.006 | 4.20E-08 | 8.14E-05 | 32 | -0.002 | 0.01 | 8.05E-01 | 0.025 | 0.018 | 1.82E-01 | 0.064 | 0.027 | 1.67E-02 |
| 1 | 167427247 | rs7523907 | C/T | 0.454 | -0.039 | 0.006 | 8.50E-10 | 1.07E-04 | 42 | -0.002 | 0.01 | 8.05E-01 | -0.013 | 0.021 | 5.28E-01 | -0.029 | 0.034 | 3.94E-01 |
| 9 | 6545605 | rs75636497 | C/G | 0.883 | 0.055 | 0.01 | 1.80E-08 | 7.67E-05 | 30 | 0.006 | 0.015 | 7.06E-01 | 0.034 | 0.031 | 2.80E-01 | -0.034 | 0.05 | 4.96E-01 |
| 1 | 198601705 | rs76181804 | A/G | 0.882 | 0.051 | 0.01 | 3.00E-08 | 6.60E-05 | 26 | -0.014 | 0.015 | 3.45E-01 | -0.025 | 0.029 | 3.95E-01 | 0.018 | 0.043 | 6.78E-01 |
| 3 | 176852038 | rs7626218 | A/T | 0.605 | 0.044 | 0.006 | 2.40E-12 | 1.36E-04 | 54 | 0.001 | 0.01 | 9.07E-01 | 0.008 | 0.021 | 7.12E-01 | 0.001 | 0.036 | 9.88E-01 |
| 10 | 9070677 | rs77000868 | T/G | 0.967 | 0.13 | 0.017 | 1.10E-14 | 1.48E-04 | 58 | 0.029 | 0.027 | 2.82E-01 | 0.105 | 0.05 | 3.36E-02 | 0.14 | 0.07 | 4.61E-02 |
| 16 | 27381648 | rs77315098 | C/A | 0.924 | 0.088 | 0.012 | 1.10E-13 | 1.36E-04 | 54 | -0.011 | 0.018 | 5.48E-01 | -0.01 | 0.036 | 7.82E-01 | -0.07 | 0.055 | 1.97E-01 |
| 12 | 121363835 | rs77741769 | C/T | 0.654 | 0.036 | 0.007 | 3.00E-08 | 6.71E-05 | 26 | -0.014 | 0.01 | 1.74E-01 | 0.004 | 0.019 | 8.15E-01 | -0.009 | 0.027 | 7.53E-01 |
| 9 | 6504189 | rs7864027 | A/C | 0.15 | 0.076 | 0.009 | 4.20E-18 | 1.81E-04 | 71 | 0.017 | 0.015 | 2.49E-01 | 0.034 | 0.032 | 2.82E-01 | 0.024 | 0.05 | 6.28E-01 |
| 7 | 28140951 | rs78823938 | G/A | 0.921 | -0.063 | 0.011 | 4.60E-08 | 8.32E-05 | 33 | 0.006 | 0.017 | 7.18E-01 | 0.015 | 0.033 | 6.48E-01 | 0.027 | 0.049 | 5.75E-01 |
| 10 | 94429467 | rs7918084 | C/T | 0.451 | -0.038 | 0.006 | 7.20E-10 | 1.02E-04 | 40 | -0.009 | 0.01 | 3.59E-01 | -0.022 | 0.021 | 2.97E-01 | -0.045 | 0.034 | 1.90E-01 |
| 3 | 33088785 | rs79337446 | C/T | 0.931 | 0.072 | 0.012 | 4.70E-09 | 9.13E-05 | 36 | 0.025 | 0.019 | 1.98E-01 | 0.023 | 0.038 | 5.35E-01 | 0.05 | 0.056 | 3.76E-01 |
| 11 | 10660840 | rs7936275 | G/T | 0.712 | -0.038 | 0.007 | 2.20E-08 | 7.47E-05 | 29 | 0 | 0.011 | 9.73E-01 | 0.023 | 0.021 | 2.61E-01 | 0.038 | 0.03 | 1.97E-01 |
| 11 | 76293726 | rs7936312 | G/T | 0.524 | -0.092 | 0.006 | 1.30E-48 | 5.96E-04 | 235 | 0.011 | 0.01 | 2.67E-01 | 0.031 | 0.019 | 1.00E-01 | 0.064 | 0.027 | 1.54E-02 |
| 17 | 37435378 | rs801429 | T/C | 0.156 | 0.053 | 0.009 | 2.60E-10 | 8.79E-05 | 35 | 0.026 | 0.014 | 7.00E-02 | 0.01 | 0.03 | 7.45E-01 | -0.048 | 0.048 | 3.19E-01 |
| 19 | 45253582 | rs8100197 | G/A | 0.727 | -0.041 | 0.007 | 3.70E-09 | 8.70E-05 | 34 | -0.008 | 0.012 | 5.26E-01 | -0.009 | 0.025 | 7.17E-01 | -0.026 | 0.039 | 5.07E-01 |
| 19 | 46370381 | rs8103278 | G/A | 0.647 | 0.036 | 0.007 | 2.00E-08 | 6.71E-05 | 26 | -0.002 | 0.011 | 8.37E-01 | -0.021 | 0.023 | 3.61E-01 | -0.052 | 0.036 | 1.44E-01 |
| 10 | 9010624 | rs827637 | A/G | 0.449 | -0.04 | 0.006 | 1.80E-10 | 1.13E-04 | 44 | -0.01 | 0.01 | 3.13E-01 | -0.007 | 0.019 | 6.94E-01 | -0.014 | 0.027 | 6.02E-01 |
| 1 | 203091274 | rs903361 | G/A | 0.341 | -0.046 | 0.007 | 9.80E-12 | 1.10E-04 | 43 | 0.008 | 0.01 | 4.16E-01 | 0.027 | 0.02 | 1.70E-01 | 0.026 | 0.027 | 3.52E-01 |
| 13 | 44485076 | rs9316058 | G/C | 0.796 | -0.043 | 0.008 | 9.80E-09 | 7.33E-05 | 29 | -0.008 | 0.011 | 4.85E-01 | 0.002 | 0.022 | 9.28E-01 | -0.035 | 0.031 | 2.71E-01 |
| 5 | 132069706 | rs9327638 | G/A | 0.804 | 0.056 | 0.008 | 1.30E-11 | 1.24E-04 | 49 | 0.001 | 0.014 | 9.48E-01 | 0.014 | 0.03 | 6.45E-01 | -0.045 | 0.049 | 3.61E-01 |
| 6 | 31326292 | rs9405085 | A/G | 0.943 | -0.091 | 0.016 | 2.10E-08 | 8.20E-05 | 32 | 0.003 | 0.018 | 8.82E-01 | -0.003 | 0.036 | 9.42E-01 | -0.014 | 0.052 | 7.88E-01 |
| 6 | 33570295 | rs9469520 | C/T | 0.754 | 0.046 | 0.007 | 2.40E-10 | 1.10E-04 | 43 | 0.006 | 0.012 | 6.43E-01 | 0.021 | 0.025 | 4.12E-01 | 0.004 | 0.044 | 9.22E-01 |
| 22 | 41908405 | rs9611602 | T/C | 0.806 | -0.048 | 0.008 | 2.90E-09 | 9.13E-05 | 36 | 0.01 | 0.012 | 3.84E-01 | 0.007 | 0.023 | 7.59E-01 | 0.044 | 0.032 | 1.69E-01 |
| 10 | 6055604 | rs9663421 | C/T | 0.724 | 0.044 | 0.007 | 2.60E-10 | 1.00E-04 | 40 | 0.007 | 0.011 | 5.17E-01 | 0.008 | 0.023 | 7.18E-01 | -0.067 | 0.038 | 7.36E-02 |
| 3 | 188045016 | rs9852988 | A/G | 0.882 | -0.053 | 0.01 | 3.20E-08 | 7.12E-05 | 28 | -0.032 | 0.014 | 2.46E-02 | -0.024 | 0.027 | 3.85E-01 | -0.074 | 0.039 | 6.02E-02 |
| 9 | 6209697 | rs992969 | A/G | 0.252 | 0.12 | 0.007 | 2.80E-64 | 7.45E-04 | 294 | 0.001 | 0.011 | 9.48E-01 | 0.017 | 0.021 | 4.40E-01 | 0.023 | 0.03 | 4.51E-01 |
| **Instrumental variables of moderate-to-severe asthma** | | | | | |  |  |  |  |  |  |  |  |  |  |  |  |  |
| 15 | 61068704 | rs10519068 | A/G | 0.128 | -0.163 | 0.023 | 1.84E-12 | 8.70E-04 | 50 | 0.006 | 0.014 | 6.34E-01 | 0.017 | 0.026 | 5.13E-01 | -0.006 | 0.039 | 8.71E-01 |
| 10 | 8115362 | rs10905284 | A/C | 0.571 | -0.105 | 0.017 | 1.76E-10 | 6.61E-04 | 38 | 0.016 | 0.01 | 1.28E-01 | 0.008 | 0.022 | 7.12E-01 | 0.019 | 0.034 | 5.74E-01 |
| 17 | 47439302 | rs112502960 | A/G | 0.359 | 0.104 | 0.016 | 4.12E-11 | 7.32E-04 | 42 | 0.007 | 0.012 | 5.56E-01 | - | - | - | -0.042 | 0.037 | 2.63E-01 |
| 11 | 1136478 | rs11603634 | G/A | 0.504 | 0.086 | 0.015 | 2.32E-08 | 5.69E-04 | 33 | 0.007 | 0.01 | 5.08E-01 | 0.005 | 0.021 | 7.97E-01 | -0.035 | 0.034 | 3.08E-01 |
| 2 | 102949161 | rs12479210 | T/C | 0.387 | 0.174 | 0.015 | 1.57E-29 | 2.33E-03 | 135 | -0.005 | 0.01 | 6.02E-01 | -0.009 | 0.019 | 6.39E-01 | 0.028 | 0.027 | 3.05E-01 |
| 17 | 47448346 | rs12952581 | A/G | 0.359 | 0.104 | 0.016 | 4.12E-11 | 7.32E-04 | 42 | - | - | - | -0.023 | 0.019 | 2.19E-01 | - | - | - |
| 8 | 81267236 | rs13274067 | A/G | 0.631 | -0.117 | 0.016 | 6.53E-13 | 9.26E-04 | 53 | -0.011 | 0.01 | 2.78E-01 | -0.009 | 0.019 | 6.49E-01 | 0.01 | 0.028 | 7.30E-01 |
| 5 | 110467499 | rs1438673 | T/C | 0.492 | -0.117 | 0.016 | 3.29E-13 | 9.26E-04 | 53 | -0.023 | 0.009 | 1.53E-02 | -0.012 | 0.018 | 5.17E-01 | -0.015 | 0.027 | 5.66E-01 |
| 9 | 6208030 | rs144829310 | T/G | 0.164 | 0.191 | 0.021 | 2.29E-20 | 1.43E-03 | 83 | 0.008 | 0.013 | 5.53E-01 | 0.036 | 0.025 | 1.46E-01 | 0.017 | 0.035 | 6.28E-01 |
| 6 | 91006227 | rs1504215 | A/G | 0.385 | -0.105 | 0.016 | 6.30E-11 | 7.46E-04 | 43 | -0.009 | 0.01 | 4.01E-01 | 0.009 | 0.02 | 6.57E-01 | 0.024 | 0.029 | 3.99E-01 |
| 5 | 110401872 | rs1837253 | C/T | 0.742 | 0.174 | 0.018 | 1.95E-22 | 1.62E-03 | 93 | 0 | 0.011 | 9.97E-01 | 0.011 | 0.021 | 6.15E-01 | -0.004 | 0.03 | 9.02E-01 |
| 5 | 131887986 | rs1986009 | A/C | 0.187 | 0.157 | 0.02 | 2.43E-15 | 1.07E-03 | 62 | -0.013 | 0.013 | 3.21E-01 | -0.004 | 0.023 | 8.50E-01 | 0.001 | 0.034 | 9.73E-01 |
| 17 | 37910368 | rs2941522 | T/C | 0.483 | 0.104 | 0.015 | 2.32E-12 | 8.33E-04 | 48 | -0.011 | 0.009 | 2.54E-01 | -0.027 | 0.018 | 1.37E-01 | -0.068 | 0.026 | 1.04E-02 |
| 2 | 242698640 | rs34290285 | A/G | 0.257 | -0.174 | 0.018 | 2.24E-23 | 1.62E-03 | 93 | 0.009 | 0.011 | 4.18E-01 | 0.016 | 0.021 | 4.52E-01 | -0.003 | 0.03 | 9.30E-01 |
| 5 | 131799626 | rs3749833 | C/T | 0.261 | 0.131 | 0.017 | 5.60E-14 | 1.03E-03 | 59 | 0.012 | 0.011 | 2.78E-01 | -0.009 | 0.021 | 6.66E-01 | -0.035 | 0.032 | 2.65E-01 |
| 6 | 32580617 | rs3997872 | A/T | 0.852 | -0.174 | 0.021 | 2.61E-16 | 1.19E-03 | 69 | 0.047 | 0.013 | 3.29E-04 | 0.052 | 0.026 | 4.22E-02 | 0.08 | 0.038 | 3.58E-02 |
| 10 | 9043404 | rs61840192 | A/G | 0.427 | -0.163 | 0.016 | 8.33E-25 | 1.80E-03 | 104 | -0.022 | 0.011 | 4.36E-02 | -0.042 | 0.022 | 6.08E-02 | -0.025 | 0.034 | 4.60E-01 |
| 12 | 57497005 | rs703816 | C/T | 0.434 | 0.113 | 0.016 | 3.69E-13 | 8.64E-04 | 50 | -0.006 | 0.01 | 5.33E-01 | 0.003 | 0.021 | 8.77E-01 | -0.002 | 0.036 | 9.63E-01 |
| 16 | 11230703 | rs7203459 | C/T | 0.246 | -0.151 | 0.017 | 4.37E-18 | 1.37E-03 | 79 | 0.005 | 0.011 | 6.43E-01 | -0.016 | 0.021 | 4.34E-01 | -0.01 | 0.03 | 7.37E-01 |
| 4 | 123158141 | rs72687036 | G/A | 0.236 | 0.113 | 0.019 | 3.06E-09 | 6.13E-04 | 35 | -0.017 | 0.011 | 1.44E-01 | -0.019 | 0.022 | 3.80E-01 | -0.015 | 0.033 | 6.57E-01 |
| 15 | 67441750 | rs72743461 | A/C | 0.236 | 0.131 | 0.017 | 4.52E-14 | 1.03E-03 | 59 | 0.002 | 0.011 | 8.67E-01 | -0.029 | 0.022 | 1.73E-01 | -0.061 | 0.032 | 5.44E-02 |
| 1 | 167427247 | rs7523907 | T/C | 0.541 | 0.095 | 0.016 | 4.82E-09 | 6.11E-04 | 35 | 0.002 | 0.01 | 8.05E-01 | 0.013 | 0.021 | 5.28E-01 | 0.029 | 0.034 | 3.94E-01 |
| 11 | 76293726 | rs7936312 | T/G | 0.474 | 0.157 | 0.016 | 6.18E-24 | 1.67E-03 | 96 | -0.011 | 0.01 | 2.67E-01 | -0.031 | 0.019 | 1.00E-01 | -0.064 | 0.027 | 1.54E-02 |

Chr, chromosome; EA, effect allele; OA, other allele; EAF, allele frequency of effect allele; SE, standard error of beta; SampleSize, available sample size for each variant in according studies; R^2^, variance in exposure explained by each variant, calculated as: R²=2*Beta^2*EAF*(1-EAF)/(2*Beta^2*EAF*(1-EAF)+ 2*SE^2*SampleSize*EAF*(1-EAF))[4]; F-statistic calculated as: F-statistic=R²*(SampleSize-2)/(1-R²)[5].

REFERENCE

[4] Gill, D., Efstathiadou, A., Cawood, K., Tzoulaki, I., Dehghan, A. (2019). Education protects against coronary heart disease and stroke independently of cognitive function: evidence from Mendelian randomization. *Int J Epidemiol.* 48, 1468-1477. doi: 10.1093/ije/dyz200.

[5] Palmer, T. M., Lawlor, D. A., Harbord, R. M., Sheehan, N. A., Tobias, J. H., Timpson, N. J., et al. (2012). Using Multiple Genetic Variants as Instrumental

Variables for Modifiable Risk Factors. *Stat. Methods Med. Res*. 21, 223-242. doi:10.1177/0962280210394459
