# Supplementary material for: Non-Causal Effects of Asthma on COVID-19 Susceptibility and Severity
Source: Front Genet. 2022 Jan 10;12:762697. doi: 10.3389/fgene.2021.762697 (PMC8784851; doi:10.3389/fgene.2021.762697)
Supplement: Supplementary file 8 [file Table3.docx]

**Supplementary Table 3.** Power calculation for the association estimates in Mendelian randomization analysis

| **Exposure** | **Outcome** | **Estimated R^2^ for exposure (%)** | **Participants in outcome GWAS data (proportion of cases, %)** | **Power to detect OR of following magnitude** | | |
| --- | --- | --- | --- | --- | --- | --- |
|  |  |  |  | **1.06** | **1.09** | **1.25** |
| Asthma | Susceptibility | 2.35 | 1 683 768 (2.315) | - | 0.80 | - |
| Asthma | Hospitalization | 2.27 | 1 887 658 (5.290) | 0.80 | - | - |
| Asthma | Severe disease | 2.34 | 1 388 342 (0.367) | - | - | 0.80 |
| Moderate-to-severe asthma | Susceptibility | 2.45 | 1 683 768 (2.315) | - | 0.80 | - |
| Moderate-to-severe asthma | Hospitalization | 2.45 | 1 887 658 (5.290) | 0.80 | - | - |
| Moderate-to-severe asthma | Severe disease | 2.45 | 1 388 342 (0.367) | - | - | 0.80 |
